# Hough Transform Implementation For Event-Based Systems: Concepts and Challenges

**DOI:** 10.3389/fncom.2018.00103

**Published:** 2018-12-21

**Authors:** Sajjad Seifozzakerini, Wei-Yun Yau, Kezhi Mao, Hossein Nejati

**Affiliations:** ^1^Institute for Infocomm Research, Agency for Science, Technology and Research (A*STAR), Singapore, Singapore; ^2^School of Electrical and Electronic Engineering, Nanyang Technological University (NTU), Singapore, Singapore; ^3^Information Systems Technology and Design (ISTD), Singapore University of Technology and Design (SUTD), Singapore, Singapore

**Keywords:** Hough Transform (HT), dynamic vision sensor (DVS), parameter space, spiking neural network (SNN), inhibitory connections, event-based video, line segment detection (LSD), generalized Hough transform (GHT)

## Abstract

Hough transform (HT) is one of the most well-known techniques in computer vision that has been the basis of many practical image processing algorithms. HT however is designed to work for frame-based systems such as conventional digital cameras. Recently, event-based systems such as Dynamic Vision Sensor (DVS) cameras, has become popular among researchers. Event-based cameras have a significantly high temporal resolution (1 μs), but each pixel can only detect change and not color. As such, the conventional image processing algorithms cannot be readily applied to event-based output streams. Therefore, it is necessary to adapt the conventional image processing algorithms for event-based cameras. This paper provides a systematic explanation, starting from extending conventional HT to 3D HT, adaptation to event-based systems, and the implementation of the 3D HT using Spiking Neural Networks (SNNs). Using SNN enables the proposed solution to be easily realized on hardware using FPGA, without requiring CPU or additional memory. In addition, we also discuss techniques for optimal SNN-based implementation using efficient number of neurons for the required accuracy and resolution along each dimension, without increasing the overall computational complexity. We hope that this will help to reduce the gap between event-based and frame-based systems.

## 1. Introduction

Neuromorphic engineering is an inter-disciplinary field focusing on implementing the biological neural systems on software and hardware systems such as analog, digital, or mixed-mode electronic circuits. This implementation can be done at different levels. For example, in the area of human vision system, the implementation can be for a simple neuron, many neurons as a vision sensor, the visual cortex or even the whole brain (Smith, 2010).

Izhikevich (2004) introduces many models of spiking neurons and compares them with respect to biological plausibility and cost of implementation. 20 different neuro-computational properties are considered for a model to be biologically plausible. The author concludes that the best model to be used depends on the application. If the purpose is to study a real neuron behavior under specific conditions, Hodgkin–Huxley model is recommended as it is the most biologically plausible. However, its computation complexity is very high. On the other hand, if the goal is simulating a large number of neurons in real time, the most appropriate model is the Integrate-and-Fire (IF) model as it is the most computationally efficient. However, it has low similarity to real neurons.

Spiking Neural Network (SNN) is the third generation of Artificial Neural Network (ANN) models. Compared to the conventional ANN, SNN is more biologically plausible since it incorporates spike times into computational models which mimic the information processing in the biological neural system. Recent advances in VLSI technology have solved the spiking neurons' implementation issues (Painkras et al., 2013; Merolla et al., 2014) that we faced previously because of their higher computational complexity compared to conventional artificial neurons, and thus have largely boosted the research and development of SNN. SNN has been used for many tasks such as learning (Ponulak and Kasiński, 2010; Yu et al., 2013a,b) and classification (Chen et al., 2012; Hu et al., 2013; Zhao et al., 2015). Among the various spiking neuron models proposed in the literatures (Izhikevich, 2003; Gütig and Sompolinsky, 2006), the most popular one is Leaky Integrate-and-Fire (LIF) neuron model (Burkitt, 2006a) due to its simplicity. Since the output of DVS can be considered as some spikes in time, it is highly consistent with any SNN input and combination of them has been a trending research interest.

Dynamic Vision Sensor (DVS) is one of the latest Neuromorphic implementation of a real visual system. Currently, they are available in two different resolutions 128 × 128 (Lichtsteiner et al., 2006, 2008; Leñero-Bardallo et al., 2011; Serrano-Gotarredona and Linares-Barranco, 2013) or 240 × 180 (Berner et al., 2013; Brandli et al., 2014). Conventional cameras are frame-based systems, i.e., capturing a series of frames which have information about all the pixels. Therefore, there is a lot of information in each frame, and most of that is redundant. The high volume of information in each frame prevents the camera to have a better time resolution (Posch et al., 2014). On the other hand, DVS captures only the changes in pixels intensity and as a result, it generates less redundant data. This data is transmitted serially using Address Event Representation (AER) protocol. Moreover, due to the change of logarithmic intensity detection, DVS offers a very high-dynamic range, meaning it has no problem in capturing the scenes which contain both very dark and very bright areas (Lichtsteiner et al., 2006).

Although DVS devices are relatively new, they have many applications in machine vision, robotics, high-speed tracking and surveillance. Benosman et al. (2012), Benosman et al. (2014) and Clady et al. (2014) have introduced some methods for event-based visual flow extraction. They are accurate in detecting the normal velocity of the objects in DVS videos. The corner points of an object are the intersecting points of two edges in different directions. For a corner point, the velocity components along the perpendicular directions to two intersecting edges are extracted. These components can be used to recognize the corner points and their actual velocity (Clady et al., 2015). In another work, a tracking algorithm (Zhao et al., 2012) has been suggested based on a Gaussian modeling of objects (Lagorce et al., 2015). Subsequently, this algorithm has been generalized for part-based shapes by minimizing an energy function defined by the parts' location and distance (Valeiras et al., 2015). Bauer et al. (2007) used a DVS as a traffic camera to track a vehicle and estimate its velocity. Due to DVS's high temporal resolution, several event-based algorithms have been introduced for tracking objects such as circles with varying diameters (Delbruck and Lichtsteiner, 2007; Delbruck and Lang, 2013) and micro-particles (Ni et al., 2012). Other applications of DVS proposed include robot self-localization (Weikersdorfer and Conradt, 2012; Weikersdorfer et al., 2013; Clady et al., 2014), terrain map reconstruction (Brandli et al., 2013), object recognition (Neftci et al., 2013; Pérez-Carrasco et al., 2013; Zhao et al., 2015) and gesture recognition (Kohn et al., 2012).

Hough Transform (HT) is a well-known method in computer vision to efficiently identify lines in images. The first appearance of HT was in a patent application by Paul V C Hough in 1962, for machine analysis of bubble chamber photographs (Hough, 1962) with the name “Method and Means for Recognizing Complex Patterns.” Then, in 1972, HT was proposed as a feature extraction (especially line detection) method in computer vision (Duda and Hart, 1972). Since then, it has been used in a wide range of pattern recognition applications in more than 2500 research articles. The main idea of this method is to first transform every point from the conventional Cartesian coordinates to the Hough parameter space (or parameter space in short), where every point defines a specific shape. Therefore, finding the local maxima through a voting procedure in the parameter space is equivalent to obtaining the shape parameters. The dimension of the parameter space depends on the shape that is to be extracted and its complexity. For example, a line can be uniquely defined by two parameters and therefore the parameter space for detecting lines is two dimensional. On the other hand, three parameters (x and y positions of centroid and radius) can define a circle on a plane, and thus the parameter space for detecting circles is three dimensional. HT can also be used for detecting arbitrary shapes (Ballard, 1981). Shen and Wang (2002) proposes a quick method for corner point detection. They detect simple lines which pass through the local coordinate origin. As a result, these lines can be expressed by only one parameter leading to a one-dimensional Hough Transform which is much faster. Moreover, they use both gradient magnitude threshold and gray level analysis to reduce the effect of noise. Jung and Schramm (2004) introduces a method for rectangle detection. Hough transform is implemented on a sliding disk-shape window over the frame. The internal diameter of the disk should be smaller than the short edge of the rectangle while the external diameter of the disk should be larger than the diameter of the rectangle. Each edge of the rectangle is identified as a peak point in parameter space. These points should satisfy some geometric conditions if they belong to a rectangle. Kang et al. (2005) presents another method for corner detection. Hough transform is used to find line segments in the frame and then inverse Hough transform is utilized to calculate the intersection points in Cartesian space. Bruckmann et al. (2014) proposes a SNN based on the HT for 2D slope and sinusoidal shape detection. After a training stage, the network is able to discriminate among different test patterns. Bachiller-Burgos et al. (2018) presents a 3D Hough transform model for detecting corner points using a 3D SNN. In addition to *r* and θ which are the normal parameters of Hough transform, the position of the line segment on a particular orientation is used as the third parameter to create the third dimension. The neurons receive cumulative excitation from not only their respective line segment but also their neighboring line segment on the same orientation in the Cartesian space. Although our proposed algorithm here uses a similar SNN, the voting procedure is different. Moreover we aim to present a general procedure and not a restricted version for a particular task.

Here, we aim to develop a systematic approach for free-form shape detection using Hough Transform and its implementation suitable for event-based systems. The key contributions are:

- Event based implementation of Hough Transform in Spiking Neural Networks
- Method to suppress redundant lines using inhibitory connections
- Extended 3D Hough Transform implementation with the following capabilities:
  - Detecting start and end points of line segments even if they are located on a same orientation which is still lacking in the conventional 2D Hough Transform
  - Removing effect of noisy events which is a common problem in 2D Hough Transform
  - Suppressing redundant lines using inhibitory connections applicable for 3D Hough Transform
- A novel non-linear parameter space quantization in extended 3D hough transform, to optimize the number of neurons in the network

We focus our discussion on implementation of Hough Transform via a two- or three-dimensional SNN to find all locally linear elements in a video captured in an event-based input stream (e.g., from a DVS camera). These elements can be then utilized to obtain shape information of a free-form curve, and to run other possible post-processing steps for shape encoding, extraction, etc. We start with brief details of DVS camera as our capturing hardware (event-based input stream), and continue to discuss the necessary details of Hough Transform and its concepts and challenges. Subsequently, we describe SNNs and its implement of 2D and 3D Hough Transform and the associated parameters to be considered for each application, including neuron potential threshold and neuron potential decay rate. Next, we discuss in details about the various types of temporal and spacial inhibitory connections and their effects and its use to suppress noise. Finally, we discuss in detail uniform and non-uniform parameter space quantization and their relation to different application settings followed by conclusion and future works.

## 2. Dynamic Vision Sensor

Dynamic Vision Sensor (DVS) is a recent neuromorphic device mimicking human visual system. The main characteristic of DVS is its high temporal capturing resolution (up to 1 microsecond). These cameras are event-based; meaning that when there is a variation in the intensity of a pixel, a polarized event is created in the form of a vector. The vector of each event has three elements (*x, y, t*); The coordinate (*x, y*) defining position of the pixel while the term *t* showing time of the event. In addition, the event polarization represents the change direction of the pixel intensity.

**Figure 1:**
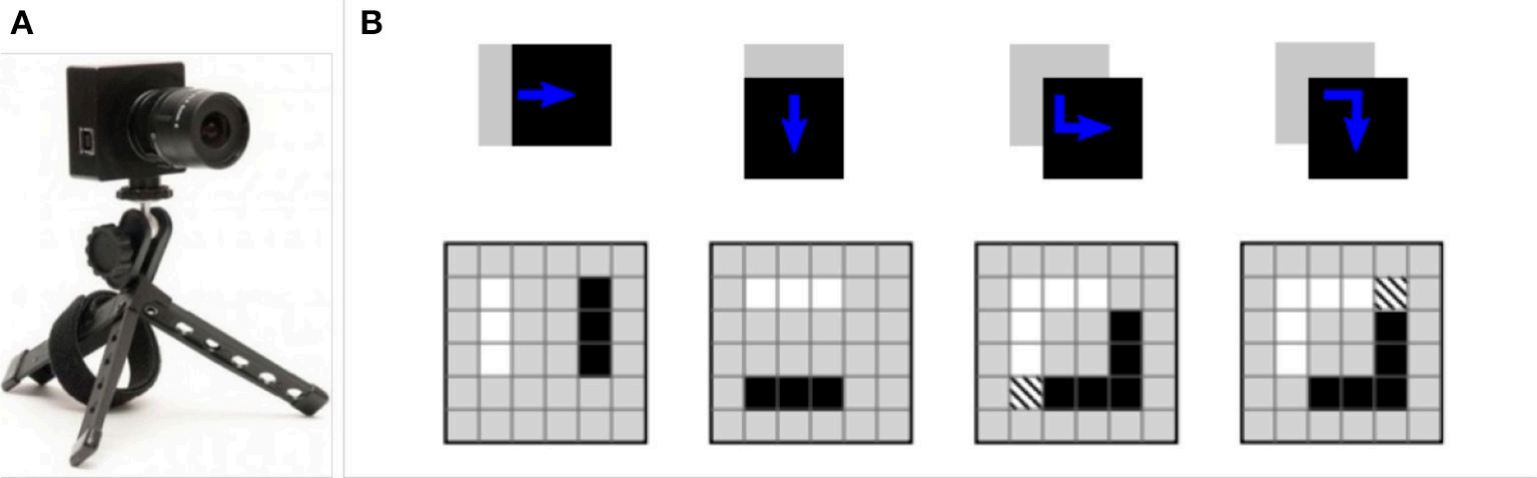
**(A)** A DVS camera, **(B)** The generated events by a moving simple square (Scaramuzza and Floreano, 2014).

Figure 1 shows an example of DVS and how it operates for a very simple shape. Let us suppose a black square is moving from left to right on a white background. When the motion is horizontal, two parallel vertical lines of events occur along the left and right edges of the square. In contrast for the vertical movement, lines of the events are horizontal, along the top and bottom edges of the square. Note that black pixels show negative events while white pixels show positive events. For diagonal motion, a complete square of events occur. The hashed pixel represents a positive event following a negative one (Scaramuzza and Floreano, 2014). According to the explanation, DVS usually captures the boundary of objects and outputs bipolar events from which edge information can be extracted. In other words, the output of DVS is directly compatible with Hough Transform without any further stage of edge detection which is necessary for conventional images.

The DVS we use in this study has a latency of 15μ*s* in hardware level. However, events are received with more delay because of an USB interface mechanism which sends data in blocks. The USB delay does not affect the content of DVS output data, and therefore, the accuracy of the received data from DVS is assured (Lichtsteiner et al., 2008).

**Figure 2:**
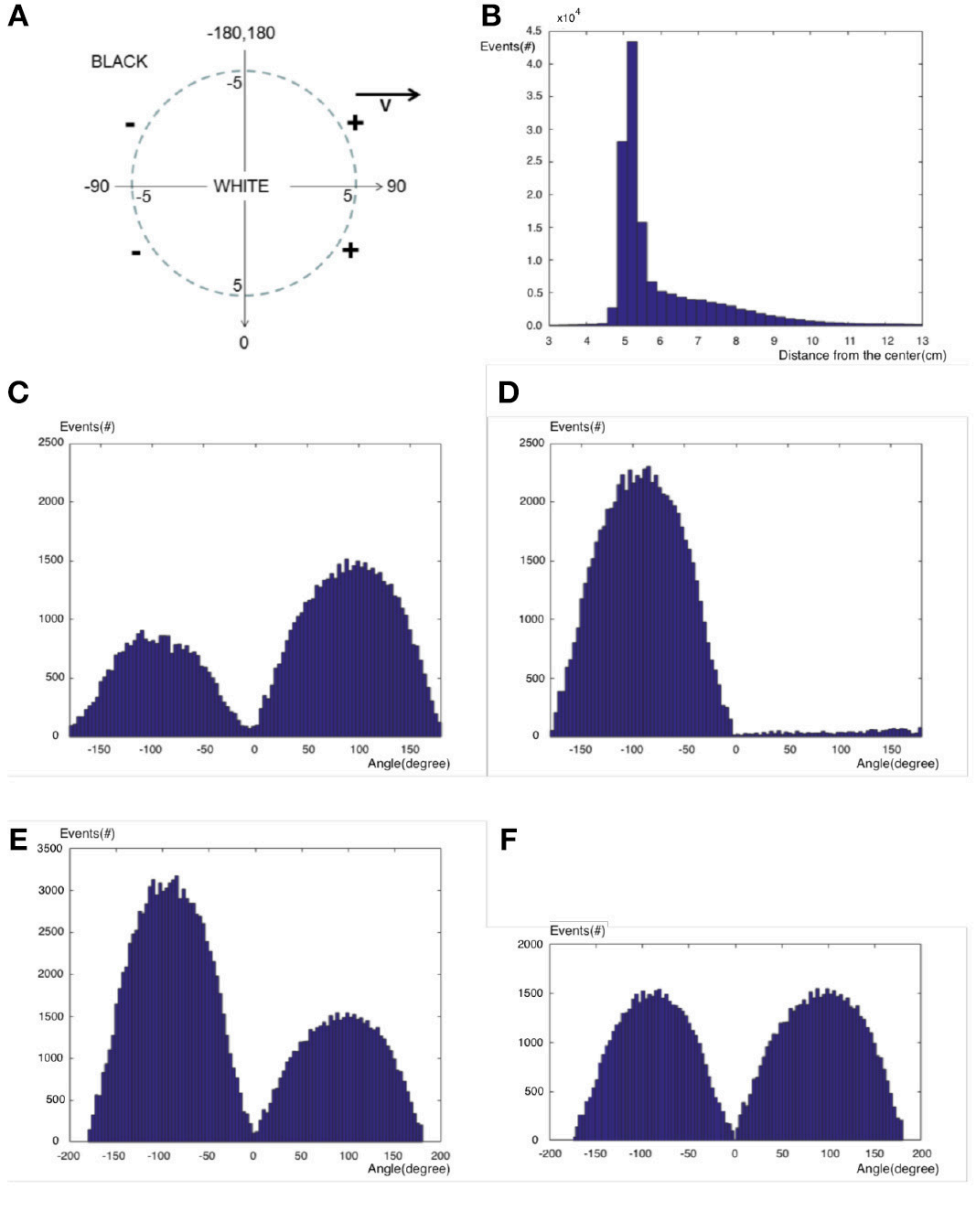
**(A)** A moving circle and expected events, **(B)** Distribution of all events over different distances from the origin, **(C)** Distribution of positive events over different angles, **(D)** Distribution of negative events over different angles, **(E)** Distribution of all events over different angles not considering their polarization, **(F)** Distribution of all events over different angles considering their polarization.

Let us investigate the pattern of generated events for a moving circle at the velocity of 10*cm*/*s*. A video of a white circle with the radius of 5*cm* is generated on a black background which is moving from the left to the right side on the horizontal mid line of the screen. This video is played in 100*FPS*, and the video is captured by a DVS while the transformation matrix is known from real coordinates to camera coordinates based on the camera calibration procedure. If the origin is fixed on the circle center which is moving horizontally from left to right, we expect positive/negative events at the right/left half of the circle ([0^*o*^, 180^*o*^]/[−180^*o*^, 0^*o*^]) as seen in Figure 2A. By receiving any event, its distance from the circle center, as well as its angle with respect to the vertical axis of real coordinates, are calculated and stored in the event vector.

Figure 2B shows the distribution of events distances from the circle center for all events. Since the circle radius is 5*cm*, the events are mostly located at the distance of 5*cm* from the circle center. They have exponential-like distribution (λ*e*^−λ*r*^) around the boundary both inside and outside of the circle. It is observed that the exponential distribution inside the circle (brighter side) has a larger parameter λ than the exponential distribution outside the circle (darker side).

Figure 2D shows the distribution of angles of events with respect to vertical axis only for negative events. Negative events are mostly located at the left half of the circle as we expected. Figure 2C shows the distribution of angles of events with respect to vertical axis only for positive events. Contrary to a perfect system, positive events are located at both sides of the circle, with the number of positive events exceeding number negative events.

Figure 2E shows the distribution of events angles with respect to the vertical axis for all events including negative and positive events. Ignoring the polarization of events, the left edge of the circle generates more events than the right edge. Other experiments also verify that in DVS cameras, high to low transition of intensity generates more events than low to high transition. It is observed that low to high transition generates only positive events while high to low transition generates both negative and positive events although the negative events are dominant. If the polarization of events is considered such that any positive event cancels a negative event at the left side of the circle, the remaining negative events at the left side will be as many as positive events at the right side of the circle as seen in Figure 2F. This figure also verifies that the events have a cosine-like distribution over the circle boundary which is proportional to the normal velocity of the circle. The velocity is proportional to the displacement of the object, equal to the number of pixels spanned over by the movement of the object. Therefore, this cosine-like distribution shows that DVS has a uniform distribution of events over the Cartesian space.

## 3. Hough Transform

Hough Transform (HT) is useful in pattern recognition and image processing as man-made objects have a straight line or circular profile. Examples include buildings, tables, cars, machines, discs, coins, buttons, biscuits etc. Moreover, the oblique projection of these objects into 2D space can be used for orientation estimation, as the projections of circles result in an elliptical pattern. The parameterization of circle used in Hough (1962); Duda and Hart (1972) is:

(1) $$\begin{array}{r} f_{c}\left(x,y\right)={\left(x-a\right)}^{2}+{\left(y-b\right)}^{2}-r^{2}=0 \end{array}$$

where (*a, b*) denote the center and r is the radius of the circle. Thus, each pixel in the image plane is transformed to a cone in a 3-D parameter space, also known as the Circle HT (CHT) (Kerbyson, 1995). It can be shown that by coding orientation and distance information as a complex phase, one can improve position accuracy (Kerbyson, 1995).

The general equation of an ellipse is:

(2) $$\begin{array}{r} x^{2}+b^{\prime }y^{2}+2d^{\prime }xy+2e^{\prime }x+2g^{\prime }y+c^{\prime }=0 \end{array}$$

where *b*′, *c*′, *d*′, *e*′, *g*′ are constant coefficients normalized with respect to the coefficient of *x*^2^. Based on the ellipse equation, to detect an elliptical object using HT, a 5-D accumulator array is needed, parameterizing location (co-ordinates of center), shape and orientation of an ellipse.

To further discuss the concepts involved in HT, let us focus on the detection of a straight line. Generally, we can show any line in the Cartesian space with two parameters, slope and intersection point with the vertical axis, y:

(3) $$\begin{array}{r} f\left(x,y\right)=y-mx-c=0 \end{array}$$

The slope *m* and y-intersect *c* are used to build the parameter space. i.e., any line in Cartesian space is transformed to a point in parameter space. On the other hand, any point in Cartesian space can be transformed to a line in the parameter space. These parameters are quantized in Δ*m* and Δ*c* intervals to create appropriate bins. For every pixel in Cartesian space, if it satisfies the above line equation, the vote of the corresponding bin of (*m, c*) in parameters space is incremented by one. Finally, those bins exceeding a critical threshold are found as representing straight lines in Cartesian space.

This method of line parameterization has a problem that the slope *m* as well as the y-intersect *c* of the line can be any numbers from −∞ to +∞. As a result, we need an infinite number of bins for both parameters to cover all possible lines, even in a finite Cartesian space. As a solution we use the angle and the normal distance of the line from the origin to define a line since both are limited to particular range based on the area under examination in Cartesian space.

**Figure 3:**
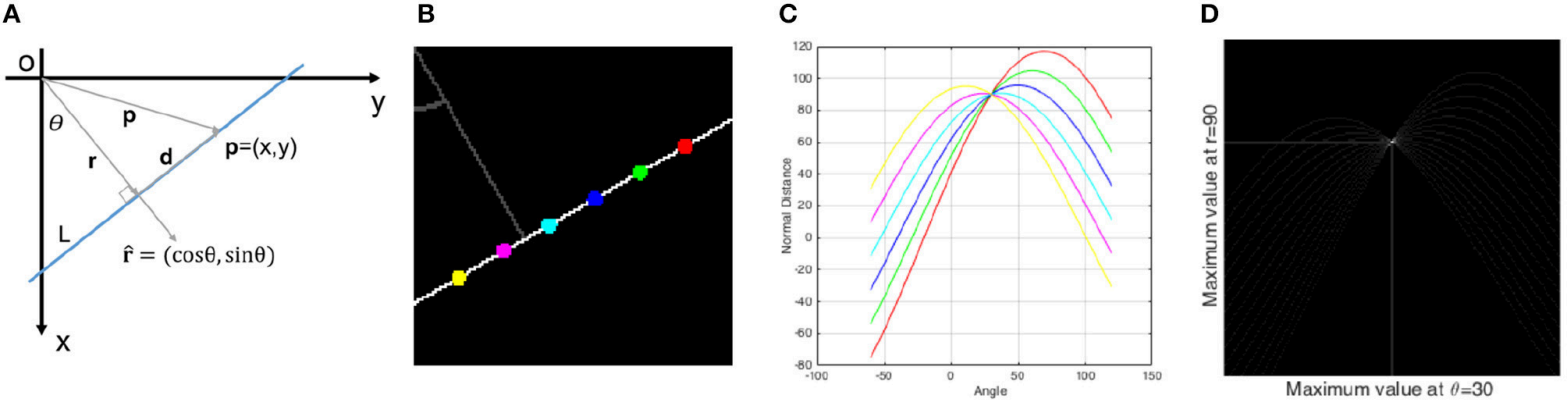
**(A)** A line in Cartesian space which is uniquely defined by two parameters *θ* and *r*, **(B)** 6 different points on a line in Cartesian space, **(C)** 6 sinusoidal curves in parameter space correspond to the 6 points, **(D)** for more points, a peak point is visible in parameter space which defines the line characteristic in Cartesian space. It is important to note that in all figures of this manuscript, *θ* is defined as the angle between the x-axis and the perpendicular distance *r*.

Let us assume a line L in Cartesian space as shown in Figure 3A. Any line in Cartesian space is uniquely defined by two parameters including the perpendicular distance *r* of the line from the origin and the angle *θ* between the perpendicular line and x-axis. The inner product of any vector **p** = (*x, y*) from origin to the line and the unit normal direction $\left[\hat{\text{r}}=\left(cos\theta ,sin\theta \right)\right]$ results in a fixed value which is *r* since the perpendicular distance of all points on the line from the origin is fixed.

(4) $$\begin{array}{r} \text{p}.\hat{\text{r}}=r\to x\cos\theta +y\sin\theta =r \end{array}$$

Equation 4 shows the transformation of any point (*x, y*) from Cartesian space to a sinusoidal curve in parameter space (*θ*, *r*). Suppose six different points on a line with six different colors as shown in Figure 3B. Any point is transformed to a sinusoidal curve as seen in Figure 3C with the same colors and all these curves pass through a common point in parameter space. The more points from line L transformed to the parameter space, the more curves pass through this intersection point as seen in Figure 3D. Therefore, there will be a point with high value (peak point) in the parameter space that can be detected by a voting procedure. The (*θ*, *r*) coordinates of this point show the characteristics of the detected line in Cartesian space.

**Figure 4:**
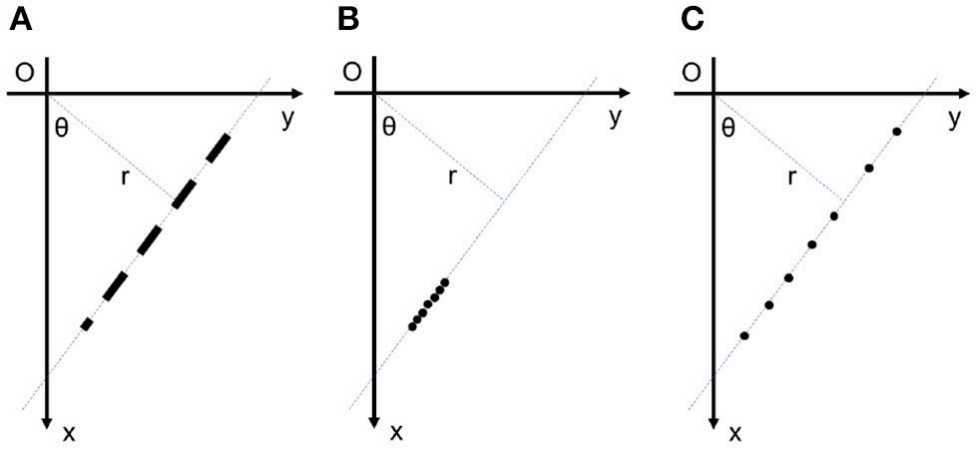
**(A)** Multiple small line segments on a direction. The standard event-based Hough transform cannot detect the position of lines on the detected direction; **(B)** 7 events from a small line segment on a direction; **(C)** 7 events on the same direction caused by noise. The standard event-based Hough transform cannot distinguish between these two cases.

Standard Hough Transform (SHT) is a powerful method that is robust against missing data and discontinuity on the curve (Hough, 1962; Duda and Hart, 1972), but SHT has several drawbacks. In SHT, computational and memory cost exponentially increases with the increase in curve parameters. Furthermore, to increase the localization accuracy in a parameter, one should increase the resolution in that parameter, i.e., more bins in quantization of the parameter, that leads to even more increase in computational and memory cost. In SHT, this quantization is done in a uniform manner, that not only require unnecessarily high memory and computation where high accuracy is not needed, it also means a non-uniform precision in detecting the curve in the Cartesian image space. Another problem with SHT is the peak spreading near the true peak, usually due to noise and other sources of error such as thick lines or lines with different gradient across their width. Furthermore, as SHT cannot detect the location of a point on the line (a.k.a blind voting), it is not possible to differentiate between points that truly belong to the line, and points that are caused by noise and happen to be on the same direction (see Figure 4 for an example). Yet again due to blind voting, if there are two or more small line segment exactly on the same direction (see Figure 4A), votes are accumulatively received for the corresponding point in all of these line segments, and while the main direction can be estimated, it is not possible to differentiate the line segments representative of different objects. Due to the same blind voting in SHT, it is not possible to automatically detect the end points of line segments.

The blind voting has yet another drawback when detecting smaller objects. As the “detection” is usually triggered based on a threshold (bins with votes more than a certain threshold are considered a hit), to detect a small object, the threshold should be also low, since such lines contain a small number of points in a frame. While lowering the threshold allows detection small line segment, it makes the detection more susceptible to noise, as SHT cannot differentiate the concentration of supporting points for a small line segment in Cartesian space, with noise distributed randomly along the same direction. To illustrate the problem better, these two scenarios are shown in Figure 4. A few points are concentrated on a direction and form a small line segment in the Figure 4B. On the other hand, the same number of points are distributed randomly on the same direction which is most likely caused by noise. If the threshold is fixed for both cases, the procedure detects a line segment in both cases, incorrectly for the second scenario.

The non-uniformity of projection of the parameter space to the Cartesian space is an intrinsic problem in the 2D HT that can severely undermine the robustness of SHT if not carefully accounted for. One main problem that plague almost all of the application of SHT in a fundamental level is the resulting non-uniformity in voting: The non-uniform projection means same-size non-overlapping spaces in the parameter space can project to non-same-size and overlapping spaces in the Cartesian space, and this leads to non-uniform vote allocation from the Cartesian space to the parameter space. Thus, it is possible that two line segments with the same size but different locations in the Cartesian frame, producing different number of votes in the parameter space, and detecting them requiring two different thresholds. Non-uniform thresholding adapted to the non-uniform projection is often ignored due to significant complexity in implementation and computational cost.

### 3.1. Generalized Hough Transform (GHT)

An initial approach toward a generalized version was made by Merlin and Farber (Merlin and Farber, 1975) by assuming the target object to be the result of translation of the model object. This idea was extended by Ballard (Ballard, 1981) into the Generalized HT (GHT), a generalization of SHT, that includes translation, rotation, and scaling of the model. GHT is a two-phase learning-detection process to detect non-parametric curves. In the first phase, learning, R-table is constructed from a model object. Then by fixing a reference point and using it as the origin, a polar co-ordinate system is established in the model object, and the R-table stores the polar coordinates of all template points. Finally, each row at the R-table is indexed by the gradient directions of the edge points on the template. In the second phase, detection, an accumulator is constructed via a 2D array, a.k.a. the Hough Counting Space (HCS) or parameter space. Matching gradients directions at each edge points and the corresponding R-table entries will add a vote to the hypothetical reference point in the HCS. The highest number of votes casted to a cell in the accumulator array (and its corresponding reference point), determines the image pattern matched to the model.

Compared to Merlin and Farber (1975), local information of object edge points is incorporated in GHT, and allow faster and more accurate execution. The local information properties were extended in Hakalahti et al. (1984), allowing application of stronger constraints for matches. These extensions include contrast, position and curvature of contour points. Further optimization of the computation is also proposed by Leavers in Dynamic GHT (DGHT) (Leavers, 1990), using available information on the distribution of feature points.

While GHT retains the robustness of SHT, it does not solve all SHT drawbacks:

- GHT cannot detect end points of line segments.
- Parallel processing of GHT requires a large number of elements.
- Brute force search is usually applied when orientation and scale of a new shape is unknown
- Due to brute force search, arbitrary shape extraction under similarity or affine transformations leads to 4D and 6D accumulator spaces, with *O*(*n*^4^) and *O*(*n*^6^) complexities, respectively.
- Unlike rigid objects, GHT cannot adequately handle flexible shapes that are usually found in nature such as leaves or animals.
- The conventional GHT cannot detect perspective transformation of planar shapes. Most images of real world objects undergo perspective transformation.

To tackle the dimensionality problem of GHT, Tsai proposed two-stage voting (Tsai, 1997). The first stage of the voting process finds the matching points with the same concavity and radii, to estimate the rotation angle of the object w.r.t the model. The second stage then matches points having the same radii, concavity and rotational angles to find the centroid of the object. Another method proposing multi-staged GHT is affine GHT in Kimura and Watanabe (2002). At the first stage, candidate points are selected by applying 2D HT. At the subsequent second stage, a 4-D HT is applied to determine the remaining four parameters. Moreover, Adaptive HT (Illingworth and Kittler, 1987) is proposed for efficient estimation of the 4-D HT at the second stage. The same concept of two-stage GHT (Tsai, 1997), but on scale and orientation is proposed in Scale and Orientation Invariant GHT (SOIGHT) in Jeng and Tsai (1991), where the accumulator array stores scale and orientation pairs for the points.

GHT can be extended to recognize articulated objects. One approach is using reference frames at joints (Beinglass and Wolfson, 1991). Another approach is a modified GHT, HT for Natural Shapes (HNS), that updates votes of all points on a line segment (Samal and Edwards, 1997). This approach can be used for natural shape recognition. An extension to HNS is proposed in Bonnet (2002) that allows template matching from a single sample of the object.

To achieve invariance to perspective transformation, the method proposed in Lo and Tsai (1997) uses a Perspective-Reference table, to store exhaustive list of perspective transformation information of the model.

### 3.2. Dimensionality Reduction in SHT

HT performs mapping of each pixel in HT space, and is therefore a time-consuming process. Moreover, the computation required to calculate HT increases exponentially with the number of curve parameters. The main proposed solution to address high-dimensional computation is subsumed under divide-and-conquer category, and either through calculating HT in sub-images, or parameter space decomposition.

Several authors divided the original image into sub-images (Davies, 1986; Ser and Siu, 1992, 1995; Gatos et al., 1996; Olson, 1999; Achalakul and Madarasmi, 2002; Chau and Siu, 2004) and applied the divide and conquer technique. The proposed methods range from single level application of HT to the sub-images under the constraint of the curve passing through a set of pixels (Olson, 1999), or multi-level application of HT, firstly on sub-images, and then evaluating the contribution of each sub-image to the HT space of the original image (Gatos et al., 1996), or parallel running of matching sub-images of template and the image, using master-slave technique (Achalakul and Madarasmi, 2002). A performance boosting technique that can be applied to all the above sub-image-based techniques is to avoid processing of relatively empty blocks. One proposed technique is thresholding blocks based on the block gradient magnitudes, and applying HT only to those that pass the threshold (Ser and Siu, 1992). Another technique estimates the contribution of each sub-image to the HT of a specific target region, and avoids calculating HT for the ones with low contribution. Fast HT (Li et al., 1986; Koshimizu and Numada, 1990) is another example that thresholds blocks based on the votes associated with each block in contributing to the HT space, with each block divided to sub-blocks to create low to high resolution application of HT, by the required calculation for a certain special resolution in the final HT.

Parameter space decomposition is the other group of divide-and-conquer methods to overcome the problem of dimensionality. For example, for detecting a curve, instead of using full HT parameter space, Pao et al. (1992) proposed decomposition of the parameter space into translation, rotation and intrinsic spaces, and then start with searching for the matching orientation of the curve in the rotation space, followed by determining intrinsic curve parameters and translation of the curve in the transformed space.

Parameter space decomposition is a popular method for reducing the dimensionality of calculating HT, in detecting circles and ellipses. Particularly, because of the geometric properties in circle and ellipse, geometric constraints can be used to avoid exhaustive search and reduce computational complexity. The parameter space decomposition in these cases usually start with finding the center of circle or ellipse, and then a guided search for candidate points that satisfy the related geometric equation. For example, in Dyer (1983); Sanz et al. (1987), the search for center point of the target ellipse is performed using a 2D array, searching for lines joining two set of points with parallel tangent, followed by a 1D array to search for candidate points of the ellipse. This method is further developed in Wallace (1983) for partially occluded ellipses, where the dimensionality reduction is obtained at the expense of high storage space. In Wallace (1983), after finding the candidate points in the second stage, the remaining 3 parameters of the ellipse (major and minor axis length and the orientation) are estimated using the following equation:

(5) $$\begin{array}{r} x+b\prime \frac{dy}{dx}+d\prime \left(x\frac{dy}{dx}+y\right)=0 \end{array}$$

Another example of using geometric constraint is presented in Muammar and Nixon (1989); Yuen et al. (1989), where the center of ellipse is found by intersecting two lines, one line, *L*1, crossing two points on the ellipse (with non-parallel tangents), and the second line, *L*2, connecting midpoint of *L*1 and the intersection tangents of the two points on the ellipse. It can be shown that the center of the ellipse lies on *L*2.

To provide a robust detection in the presence of noise and occlusion, the symmetry in the ellipse is usually used as another geometric constraint. Methods such as in Chatzis and Pitas (1997), tackle the problem of dimensionality by firstly applying geometric symmetry constraint to group all feature points into different possible ellipses, and then following by searching these groups for sets to satisfy geometric properties of the ellipse to find the remaining 3 parameters.

Detecting circles are usually on the basis of circles being special cases of ellipses. For example, the two-staged parameter space decomposition of finding the center first and then other parameters is shown in Chan (1991), using two sets of 2D accumulators. The geometric symmetry is also used in Rad et al. (2003), applied on gradient pair vectors, and in Ioannou et al. (1999) using the geometric constraint that any chord passes through the circle center. A procedure for simultaneous detection of lines and circles using conformal geometric algebra and its implementation using FPGA is proposed in López-González et al. (2016) and Soria-García et al. (2017). Klefenz et al. (1993) and Epstein et al. (2002) also present the SNN FPGA and ASIC implementation of the Parallel Hough Transform for lines and circles with binary weights.

Other examples of parameter space decomposition are use of mean squares error (MSE) to estimate one parameter at a time (Tsuji and Matsumoto, 1978), dividing the image into sub-images by convexity of the ellipse shape (Fei et al., 2009), finding The Fuzzy Cell HT (Chatzis and Pitas, 1996) and Randomized Fuzzy Cell HT (Chatzis and Pitas, 1997).

## 4. Extended Hough Transform

To overcome the limitations of SHT, several modifications can be applied in different aspects of SHT. Some of these modifications lead to significantly distinct algorithms, including Generalized Hough Transform (GHT) (Ballard, 1981), Probability based HT (Galamhos et al., 1999), Randomized HT (Xu et al., 1990), and Monte Carlo HT (Bergen and Shvaytser (Schweitzer), 1991). Here, we discuss an extension to SHT that can address multiple SHT shortcomings at the same time.

In this extended version of SHT, we consider the third dimension of the parameter space to be the position of points on the detected direction. The position of a point on a line can be described by the distance of any point on the direction from the perpendicular line to that direction. This distance *d*, is shown in Figure 12A. The distance *d* can be considered as the cross product of vector **p** = (*x, y*) and unit normal direction $\left[\hat{\text{r}}=\left(cos\theta ,sin\theta \right)\right]$ as follows:

(6) $$\begin{array}{l} p\times \hat{r}=\left|\begin{array}{ccc} \hat{i} & \hat{j} & \hat{k}\\ x & y & 0\\ \cos\theta & \sin\theta & 0 \end{array}\right|=(x\sin\theta -y\cos\theta )\hat{k}=d\hat{k}\\ \;d=x\sin\theta -y\cos\theta \end{array}$$

**Figure 5:**
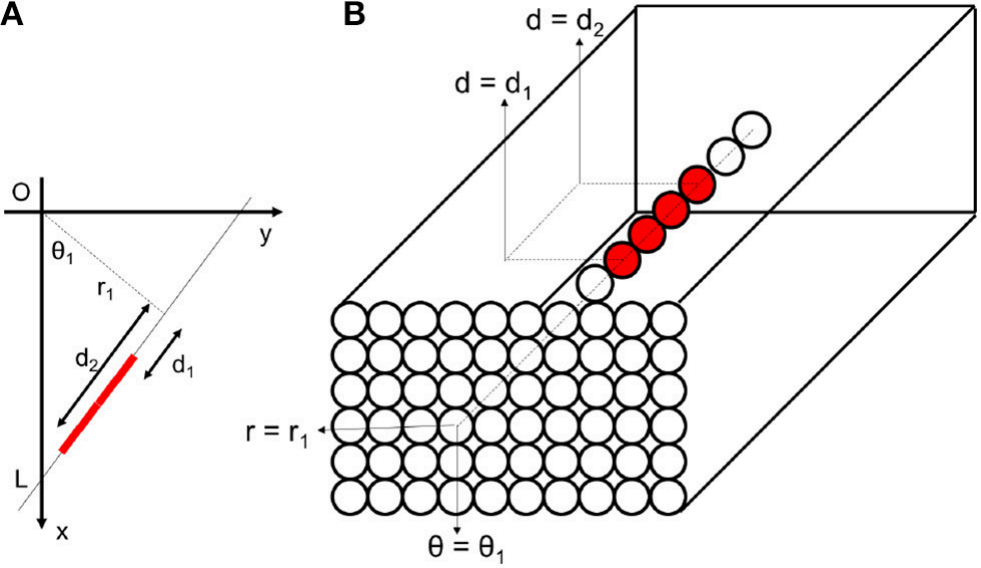
**(A)** A line (marked by red) in Cartesian space, **(B)** corresponding three-dimensional Hough area consists of different bins. Red bins represent peak points that receive more votes from Cartesian space. These bins are substituted with spiking neurons.

We can use distance *d* as the third parameter beside *r* and *θ* to build a three-dimensional Hough space as seen in Figure 5. If a Cartesian frame contains a line, in its corresponding 2D parameter space, there is a peak point at particular *r* and *θ*. In contrast, in the 3D parameter space, there are many peak points with different *d* values and at the same *r* and *θ*, as seen in Figure 5B.

Using *d* values, one can achieve a rich set of information about the shape. Firstly, based on the above definition, the right/left positions of the perpendicular line are represented by positive/negative *d* on the line (in example illustrated in Figure 3A, *d* is a negative value). Therefore, the balance of the points on the line can be achieved based on the sign of *d* values.

Furthermore, the value of *d* is also the negative derivative of *r* (perpendicular distance *r* of the line from the origin) with respect to *θ* (the angle between the perpendicular line and x-axis) based on Equation 4. In other words, the slope of curves passing through the common point in Figure 3C, shows distribution of distances of corresponding points in the Cartesian space from the perpendicular line. This property can be used in inspecting the various shape slope and balance information of the line.

**Figure 6:**
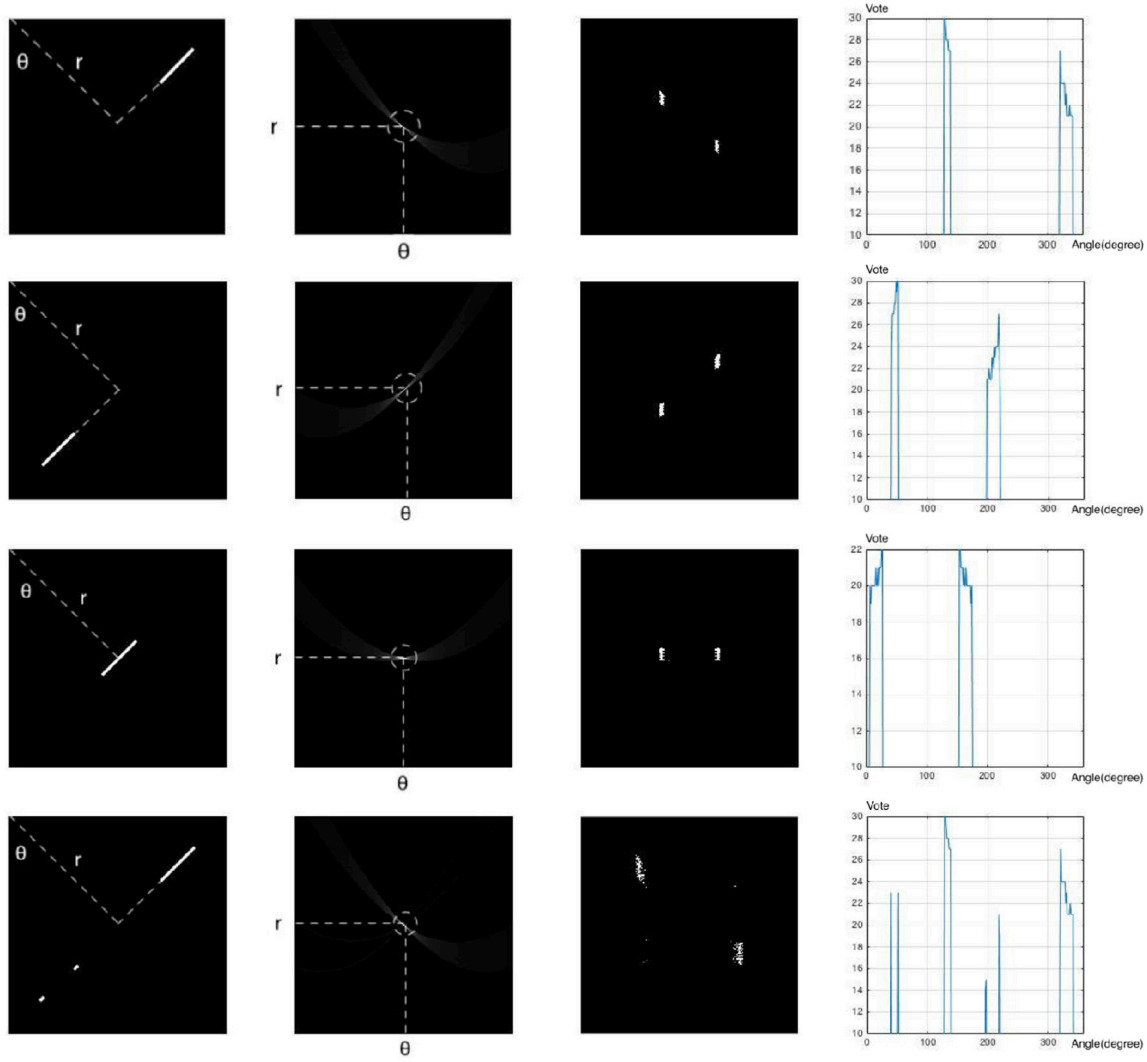
1st column: Cartesian Space; 2nd Column: Parameter Space; 3rd column: Equipotential contours in parameter space; i.e., the position of bins which receive the same number of votes (20) from the Cartesian space; 4th column: Distribution of vote values around the peak point in parameter space. The horizontal axis shows the angle and the vertical axis shows the vote values. First 3 rows represent the direction of peak spreading in parameter space for 3 different cases where the line segment is located on the left/right sides or on the perpendicular line from the origin. The last row shows the effect of noise present on the direction of the line segment.

The third dimension in the parameter space can also address the noise susceptibility in SHT when searching for smaller line segments. In order to check whether a local maximum in parameter space corresponds to a line segment, we can check the distribution of vote values around the maximum point in the third dimension. The voting values create equipotential contours around the maximum point, and also affect the sharpness of the bell-shaped peak at the maximum point. The distribution of equipotential contours reveals the distribution of points in the Cartesian space, i.e., a compact distribution corresponds to a set of points close together, more likely from a line segment, whereas a wide distribution corresponds to a set of points far from each other, more likely from random noise that are placed on the same direction by chance. This way, even smaller line segments can be easily detected as densely packed patches of high votes along the third dimension (compared with sparse distribution of votes corresponding to noise). Figure 6 illustrates these scenarios and their corresponding parameter space as well as voting distributions along the third dimension of the parameter space.

The same way, adoption of the third dimension as *d* can address the peak spreading problem. The votes are based on the distances of their corresponding points in the Cartesian space. Therefore, if due to noise or other sources of error, some points are placed in the same direction, their corresponding votes will be distributed (usually sparsely) along the third dimension, avoiding peak spreading to a great extent.

End points of line segments can also be detected by looking for sudden drop in the presence of high votes in a segment along the third dimension. The position of the drop in votes correlates with the position of the end point of the line segment in the Cartesian space.

Finally, by adopting *d* as the third dimension of the parameter space, we can overcome the major intrinsic problem of 2D SHT, non-uniformity in voting projection. As the third dimension, *d*, is uniformly projected between the Cartesian space and the parameter space, votes along this dimension are also uniformly projected between these two spaces. In other words, two line segments with the same size and direction, result in exactly the same voting patterns regardless of their placement in the Cartesian space.

## 5. Spiking Neural Network

In the brain, the communication between neurons of a neural circuit is done by sending trains of action potentials, also known as spike trains. These individual spikes are sparse in time, so each spike has high information content, and to a first approximation has uniform amplitude. Thus, information in spiking neural networks (SNNs) is conveyed by spike timing, including latencies, and spike rates, possibly over populations (Gerstner et al., 2014). SNNs almost universally use idealized spike generation mechanisms in contrast to the actual biophysical mechanisms (Hodgkin and Huxley, 1990). SNNs offer a special opportunity in energy efficiency as spike events are sparse in time. Spiking networks also have the advantage of being intrinsically sensitive to the temporal characteristics of information transmission that occurs in the biological neural systems. It has been shown that the precise timing of every spike is highly informative for several areas of the brain and plays an important role in neural coding (Gollisch and Meister, 2008). These advantages of SNNs brought these networks in the focus of a number of recent applications in many areas of pattern recognition such as visual processing (Arnold and Miklós, 2010; Meftah et al., 2010; Wysoski et al., 2010), speech recognition (Kröger et al., 2009; Wade et al., 2010; Tavanaei and Maida, 2017), and medical diagnosis (Ghosh-dastidar and Lichtenstein, 2009; Kasabov et al., 2014).

The simplest model proposed as an artificial neuron is Leaky-Integrate-and-Fire (LIF) neuron. A comprehensive review of the Leaky-Integrate-and-Fire neuron is presented in Burkitt (2006a). The synapses are considered as electrical currents into the neuron that increase the membrane potential continuously like charging a capacitor with an electrical current. The membrane potential is leaking by a fixed rate as well. When the membrane potential exceeds some threshold, the output fires and generates a spike. LIF neuron is the least complex model of a real neuron lacking many actual characteristics, but on the other hand the most suitable for hardware implementation, not requiring CPU and additional memory. Despite the simplicity of the LIF neuron, it is widely used, including explaining the control mechanism of the brain (Laing and Longtin, 2003) and network stability (Burkitt, 2006b).

**Figure 7:**
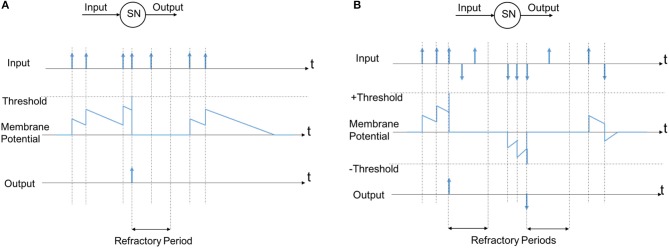
The model of a spiking neuron **(A)** normal model, **(B)** the model used in this study according to events polarization.

The main parameters in an LIF neuron are its Membrane Potential (MP) threshold and its membrane potential decaying rate. Referring to Figure 7A, every Spiking Neuron (SN) has some inputs (one input is used here for simplicity) and an output. The input is a spike train (sequence of spikes) that influences the neuron's Membrane Potential (MP). Every positive input spike causes an increase of the MP which is always decaying by a fixed rate. This rate is referred to as Membrane Potential decaying rate. Whenever the MP exceeds a certain threshold, a spike is generated in the output, the MP is then reset and the neuron enters a refractory period, during which the neuron's MP remains zero and input spikes are ignored. The firing of other SNs can also force this neuron to reset through lateral inhibition in a network. We describe the refractory period and the inhibitory connections in detail in section 6.

We can implement the Hough Transform parameter space using a spiking neural network as described in Seifozzakerini et al. (2016). While this article describes a 2-dimensional SNN (and therefore parameter space), we can extend the principles to the third dimension of HT as described in the previous chapter. The implementation of the third dimension *d* using SNN not only inherits the advantages of this new 3D HT, but also allows additional advantages including the possibility of implementation of the 3D HT with no requirement for CPU or additional memory, and efficient energy consumption, and directly using FPGAs. In addition, as the output of event-based systems such as DVS cameras are in the form of the inputs to the SNNs, the hardware implementation can be directly fed by a DVS camera, providing real time applications of image processing using the 3D HT capabilities.

Moreover, by implementing the described 3D HT using SNN, an arbitrary shape can be represented based on its locally linear components in the three dimensions, requiring no other parameterization. Based on location, length, and orientation of these small elements, important parameters like shape and curvature can be obtained and the whole shape can be extracted. With implementation of 3D HT using SNN, the complexity of the shape extractor is only governed by the number of locally linear components of the arbitrary shape, and does not increase exponentially with shape complexity, and therefore, even for extraction of an arbitrary shape, the SNN can be implemented using FPGA. While it is out of scope of this text, this information can be then used for shape encoding to be used for shape matching and object detection.

The resolution of the shape representation in this SNN-Based 3D HT is governed by the size of associated parameter space to each neuron in the SNN. As we use locally linear components, this resolution can be also freely increased along one dimension to increase the localization accuracy in that parameter, without an increase in computational complexity or dimensionality on other parameter space dimensions. Finally, by defining different classes of neurons with different associations to the parameter space, we can easily obtain a non-uniform functionality (thresholding, binning, etc.) over parameter or Cartesian spaces, without extra level of computational complexity in software or hardware implementations.

The main limitation of 3D networks is the number of neurons they need, as neurons are assigned based on the resolution of each dimension. However, the number of neurons can be reduced by 10 times, via removing neurons that do not receive any vote from the Cartesian space.

**Figure 8:**
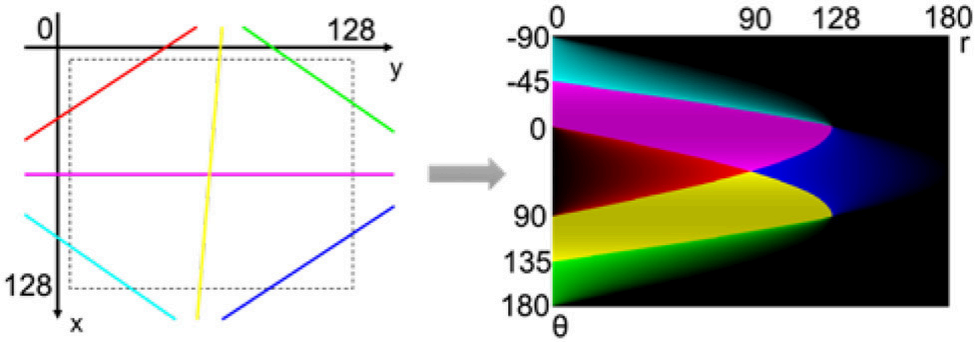
Different line positions in Cartesian space and their corresponding areas in the parameter space. The color intensity in parameter space shows the line length inside the video frame (Seifozzakerini et al., 2017).

To explain this, let's assume the 2D HT for a 128 × 128 frame. To cover all possible lines within a 128 × 128 frame, *θ* is limited between $-\frac{\pi }{2}$ and π while *r* between 0 and $128\sqrt[{}]{2}$ in the parameter space. However, due to the characteristics of Hough transform, the effective area in the parameter space is not a simple rectangle. Depending on *r* and *θ* values, the corresponding lines in the Cartesian space have variable length and different situation inside the video frame. Figure 8 shows different line positions in the video frame and their corresponding areas in the parameter space. The total area covered by all possible lines in the Cartesian frame, covers only about 60% of the rectangular frame in the parameter space, and the remaining 40% does not receive any voting. As a result, the number of spiking neurons can be reduced by 40% in hardware implementation. This reduction in the number of required neurons is even greater in 3D HT.

Note that in the 2D HT, smaller lines in the corners of the Cartesian frame generate less votes in the parameter space, illustrated by the color intensity in Figure 8 (Seifozzakerini et al., 2017). Therefore, a variable threshold should be assigned in the corresponding SNN that implements the parameter space, to account for this non-uniform voting. However, the adoption of *d* as the third dimension of the parameter space will solve this issue by assigning uniform voting from the Cartesian frame (see section 4).

Our experiment shows that about 40K neurons (instead of more than 400K) are enough to build a 3D parameter space for 128 × 128 frame in Cartesian space with Δ*r* = 3*pixels* and Δ*d* = 9*pixels*. SNNs in this scale can be easily implemented with current hardware technology.

While the LIF neuron model illustrated in Figure 7A is simple to implement, when it comes to the event-based systems, this model has a main drawback, as it ignores the polarity of DVS output events, as described in Figure 2. In section 2 we showed that considering the polarity of events can produce more robust results based on the explanation in the following. According to the observations in section 2, if we ignore the events polarization and use the spiking neuron in Figure 7A, the neurons correspond to the circle left side receive more excitation than the neurons correspond to the right side; i.e., the excitations for different neurons are imbalanced in different sides and it should be compensated by setting different thresholds for membrane potentials. A solution we use here is to utilize events polarizations in neurons excitations as shown in Figure 7B. This model is slightly different from the normal model of a spiking neuron, but it is more robust for neurons in different situations, and does not add significant complexity to the hardware implementation.

## 6. Inhibitory Connections

Neural inhibition is an active process that reduces or suppresses the excitatory activity of synapses, neurons or circuits (Martin, 2006). The concept of inhibition can be referred to interruption or blockade of activity patterns in both space and time. Proper dynamics in neuronal networks can only be maintained if the excitatory forces are counteracted by effective inhibitory forces. The dynamics between excitatory and inhibitory cells to create form or order or secure some autonomy for transiently active groups. These inhibitory cells provide the necessary autonomy and independence to neighboring principal cells. Additionally, the opposing actions of excitation and inhibition often give rise to membrane and network oscillations which, in turn, provide temporal coordination of the messages conveyed by principal cells (Jonas and Buzsaki, 2007).

**Figure 9:**
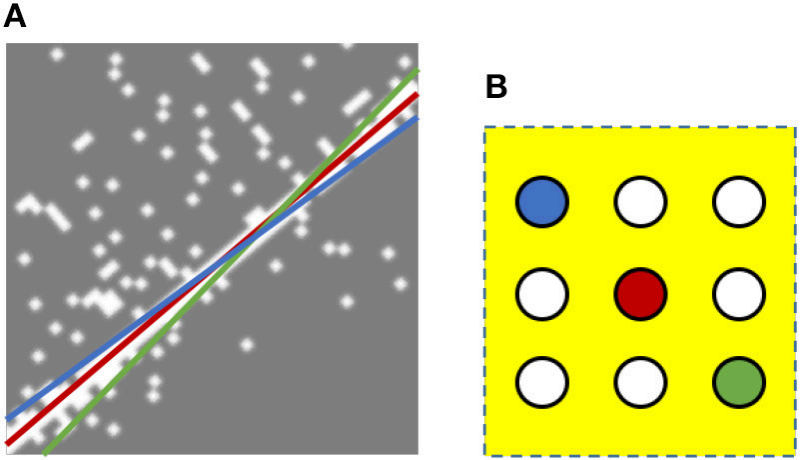
Local lateral inhibition to suppress noise lines. **(A)** Without lateral inhibition, SNN detects three lines (red, blue, and green). The best fitted one is the red line detected by the red neuron in **(B)**. The red neuron is expected to fire before blue or green ones. With local lateral inhibition, when the red neuron (the correct one) fires, it inhibits all laterally connected neurons and thus blue and green lines can be suppressed.

The same principles can be implemented in Spiking Neural Networks (SNNs) using local lateral inhibition to suppress noise (e.g., redundant lines) from being detected. This is illustrated in Figure 9 which shows the events generated by moving an edge from left to right in front of the DVS sensor. The left side of the edge is totally black while the right side is white. All events, whether positive or negative, are shown by white dots on a gray background. The detected lines using the SNN without local lateral inhibition are superimposed onto the DVS events. In Figure 9, the best fitted line, the red line, is detected by the red neuron. However, the blue and green lines also cover many events, causing blue and green neurons to fire in the parameter space. These two lines can be suppressed by the local lateral inhibition. Each neuron is laterally connected to all its neighboring neurons that are within a window around that neuron. These connections cause a local competition between the neurons inside the yellow window. Whenever the first neuron (red one in this case) fires, it resets all neighboring neurons, prevents them from firing, and thus suppresses the noise lines.

Inhibitory connections between neurons are used for suppressing lines which are close together. As seen in Figure 12C, let us suppose two different lines *E*1 = (*θ*_1_, *r*_1_, *d*_1_) and *E*2 = (*θ*_2_, *r*_2_, *d*_2_) be the best matching line elements to some existing events in Cartesian space. The corresponding neuron to the best matching line fires and the line is detected. We want to suppress all other lines which are most probably capable of being wrongly detected as redundant lines. Referring to equations 4 and 6:

(7) $$\{\begin{array}{c} r=x\cos\;\theta +y\sin\;\theta \Rightarrow \Delta r=(-x\sin\theta +y\cos\theta )\Delta \theta =-d\Delta \theta \\ d=x\sin\theta -y\cos\theta \Rightarrow \Delta d=(x\cos\theta +y\sin\theta )\Delta \theta =r\Delta \theta \end{array}$$

**Figure 10:**
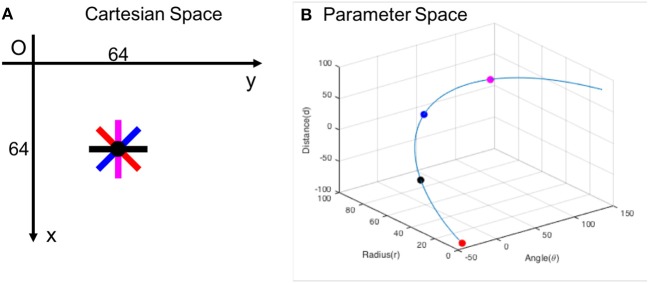
**(A)** Cartesian space and **(B)** its corresponding parameter space. Red, black, blue, magenta points correspond to line segments in the same colors in Cartesian space. Inhibitory connections are set between neurons on the blue curve in parameter space to suppress redundant lines passing through the point (64, 64) in Cartesian space.

Above equations show the mathematical relation between Δ*θ* and (Δ*r*, Δ*d*). If the objective is to suppress all redundant lines within ±Δ*θ* range in angle, the corresponding (Δ*r*, Δ*d*) are calculated based on above equations. Figure 10 shows a Cartesian space and its corresponding parameter space. Neurons on the blue curve in parameter space represent all line segments with different orientations passing through the point (64, 64) in Cartesian space, e.g., red, black, blue, magenta points correspond to line segments in the same colors in Cartesian space. Inhibitory connections are set between each neuron on the curve and its neighboring neurons on the same curve within ±Δ*θ* range in angle. In this study, we suppress all redundant lines in all directions; i.e. inhibitory connections are between each neuron on the curve and all other neurons on the same curve. It is noted that all crossing points are removed if the lines are suppressed in all directions. When a neuron fires, we find its corresponding point in Cartesian space. On this point, a resetting event is supposed, reset all affected neurons in the network.

Each output spike from SNN represents a small line segment in Cartesian space. Therefore, an inhibitory window around the line segment in the Cartesian space can be directly mapped to find the necessary inhibitory connections to a neuron in the SNN representing the parameter space. The size and shape of the inhibitory window in the Cartesian space can be defined based on the application, e.g., an application with high noisy level (larger window), or another one with high accuracy requirement (smaller window). When the parameters of this window are decided upon for a specific application, the resulting inhibitory connections can be easily implemented in an FPGA with the above method.

In event-based systems such as DVS camera, inhibition can be also applied to time, in the form of refractory period (RP) of neurons, that resets a neuron for a specific period after it fires. The extent of the RP can determine networks behavior in slow or fast pacing environments, with a longer RP preventing the network from providing false positives when the environment is slow, and shorter RP enabling the network to detect fast moving object.

## 7. Parameter Space Quantization

**Figure 11:**
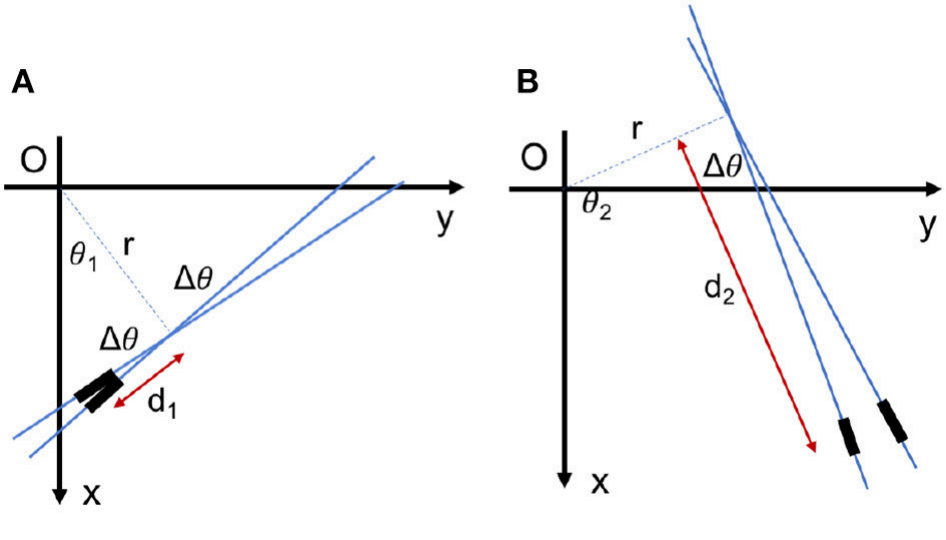
The effect of uniform quantization of parameter space. Two line elements with the same distance of Δ*θ* in Hough area, although **(A)** the elements can be near or **(B)** the elements can be far in Cartesian space.

In a real application, the parameter space should be quantized along all dimensions. Normally, a uniform quantization is applied such as in Seifozzakerini et al. (2016), due to it simplicity. But this should not mask the substantial effect of the quantization method of parameter space on the quality and accuracy of the implementation. As the HT is not a uniform projection, uniform quantization of the parameter space is not the best choice. For example, as seen in Figure 11, if a line element is located near to the perpendicular line, a small change in *θ* results in a small displacement of the line element. But the same amount of change in *θ* for the elements far from the perpendicular line causes a large displacement of the elements.

Instead, with the adoption of *d* as the third dimension, we can use a uniform quantization for *r* and *d* while the quantization of *θ* is optimized for better performance. Quantization over *d*, Δ*d*, can be at least a few times larger than, quantization over *r*, Δ*r*, since for a line segment, the length should be several times larger than the width.

**Figure 12:**
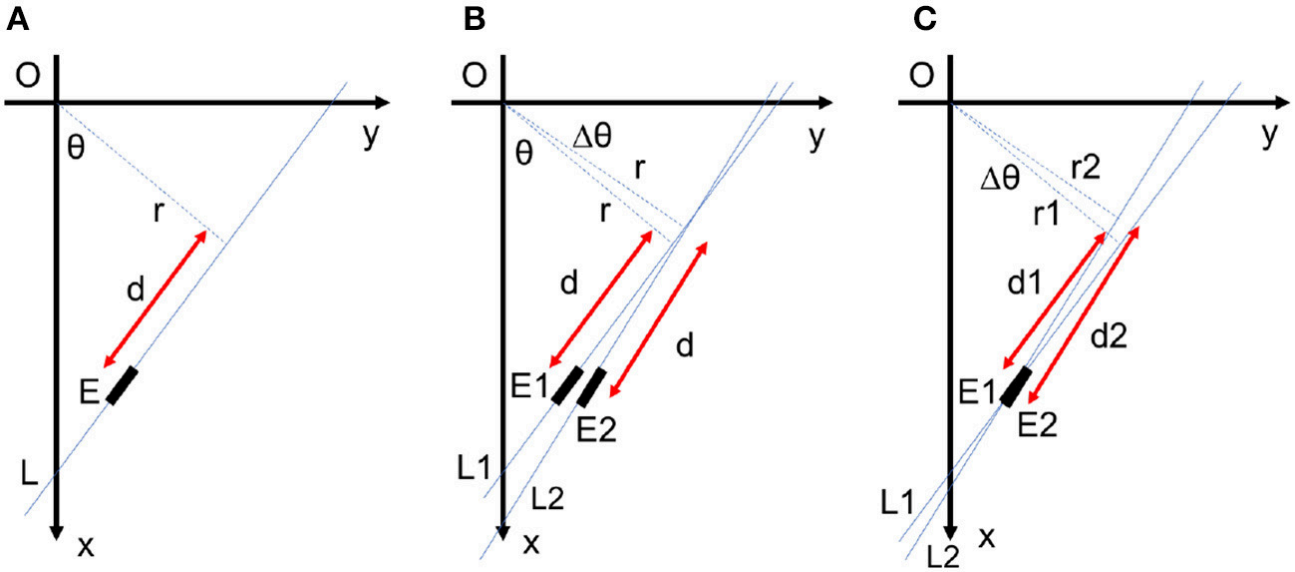
**(A)** Each line element is uniquely defined by three parameters *θ*, *r*, and *d*. **(B)** For fixed values of *r* and *d*, a small change in *θ* cause a radial displacement of *dΔ*θ** and a tangential displacement of *rΔ*θ** in the element position. **(C)** Redundant lines are located at the same place with a small difference in their orientations. These lines are suppressed by lateral inhibitory connections within the network.

Let us consider a small linear element *E*1(*θ*, *r, d*) as seen in Figure 12B. By fixing *r* and *d* of this element, a small change Δ*θ* of the angle results in a new position at *E*2(*θ*+Δ**θ*, r, d*). The displacement from *E*1 to *E*2 consists of two radial and tangential displacement. The radial part is approximately *dΔ*θ** and the tangential part is *rΔ*θ**. To cover all over the frame in Cartesian space and not to have redundant elements simultaneously, *E*1 and *E*2 should not intersect each other in the frame and there should be no space between them. As a result, the tangential displacement should be smaller than the element length and the radial displacement should be smaller than the element thickness as follows:

(8) $$\begin{array}{r} r\Delta \theta <\Delta d\Rightarrow \Delta \theta <\frac{\Delta d}{r} \end{array}$$

(9) $$\begin{array}{r} d\Delta \theta <\Delta r\Rightarrow \Delta \theta <\frac{\Delta r}{d} \end{array}$$

To find out which one of above conditions is more restrictive, we should compare $\frac{\Delta d}{r}$ and $\frac{\Delta r}{d}$ to see which one is smaller. By defining stretch ratio, $SR=\frac{\Delta d}{\Delta r}$ for line elements; if *d* is smaller than $\frac{r}{SR}$, condition 8 is more restrictive than condition 9 and vice versa. The stretch ratio is a value greater than one defined by *r* and *d* quantization which can be fixed for each application. For a specific value of *r* and *d*, if $d<\frac{r}{SR}$ the angle *θ* is quantized uniformly while for greater values of *d*, the quantization of *θ* should be finer by increasing *d*.

Based on the above parameter space quantization technique, we can avoid the non-uniform quantization of the Cartesian, as in the SHT.

Here, just as a proof of concept, we apply our extended event-based Hough Transform on 3D HT, together with SNN implementation incorporating inhibitory connections and parameter space quantization. The objective of this experiment is to show how the extended event-based Hough Transform can remove the noise and clean videos, and can be applied on any arbitrary shape e.g., circle, to recognize its boundary as a series of small lines, by automatically detecting all small line segments in the frame and removing events caused by noise.

**Figure 13:**
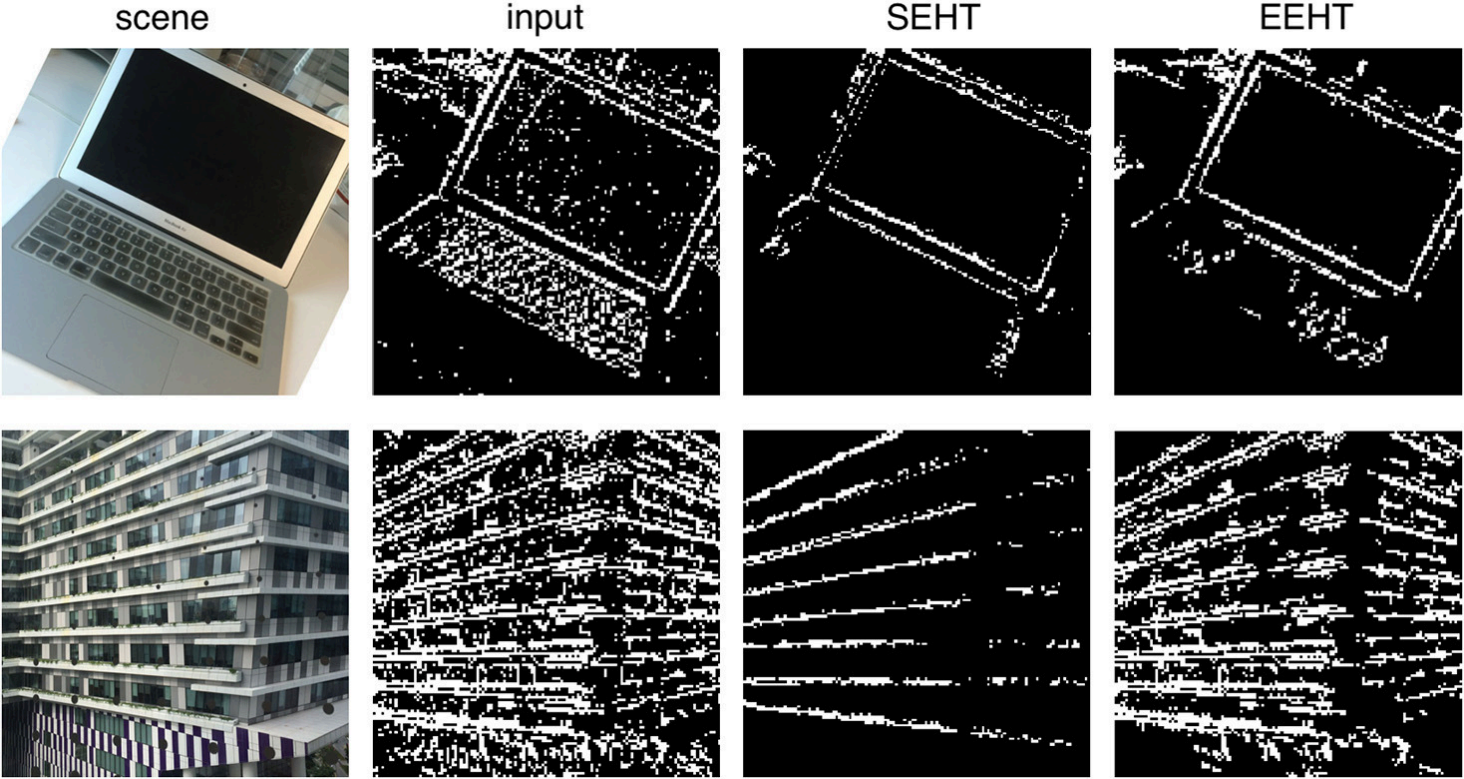
The result of Standard Event-based Hough Transform (SEHT) and Extended Event-based Hough Transform (EEHT) on two noisy scenes. The result of both algorithm contains less noise compared to the algorithm input. SEHT keeps only major directions in a scene while EEHT keeps more details. As a result, SEHT can be misleading for some streams e.g., building here.

We have chosen two noisy scenes in Figure 13 to show the capability of the algorithm in cleaning noisy videos. Please note that representing out events of an event-based system and comparing it to frame-based systems is difficult. We had to compress all the events produced in a time window, into a single frame and present it in the figures. In this way it would be easier to compare results from standard event-based HT and the extended event-based HT. Please note that all the processing for the event-based HT is performed in the original event-based space. The removal of noise has a direct relationship with the pre-selected size of the line segment. In an application where only the main structure is required, one can select larger line segment size, whereby more details would be removed from the events (e.g., removal of events associated with keys on the keyboard in Figure 13). But if the application requires details of the scene, then a smaller line segment limit should be chosen. Therefore, as the definition of noise depends on the application, the “goodness” of event removal using the proposed quantization also depends on the application. Figure 13, is only to show the concept. The exact outcome can be tuned to meet the need of the application.

**Figure 14:**
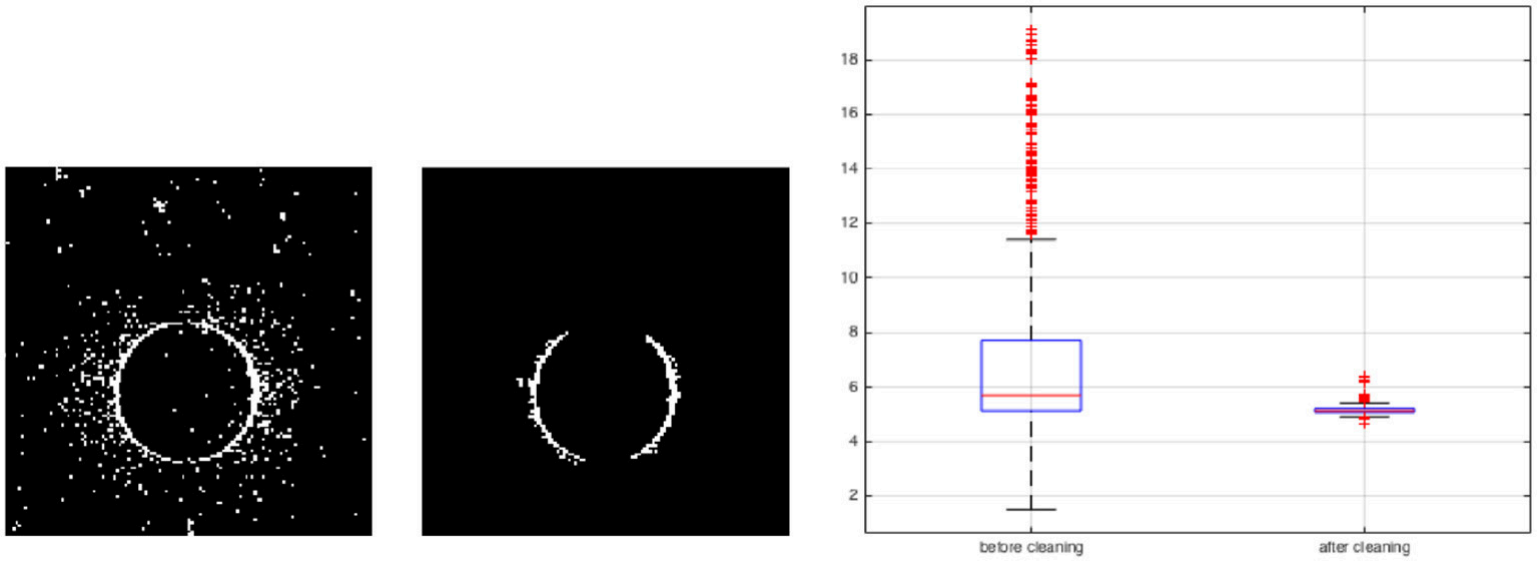
A video of a moving circle with radius of 5*cm*. The extended event-based Hough transform is applied to detect the circle as a series of small lines. Forty spikes correspond to 40 small lines are received from the spiking neural network. The result shows more narrow distribution of events around the circle boundary.

Another example is detection of a moving circle in Figure 14. A synthetic video is displayed in a 13.3-inch MacBook Air with the resolution of 1440 × 900 and aspect ratio of 16:10. Therefore the display area is 28.65 × 17.90 *cm*^2^ and there are about 50 pixels per centimeter. The synthetic video shows a white circle of radius 5cm moving horizontally from left to right on a black background with the velocity of 10*cm*/*s* and passes the center point of the screen, with a frame rate of 100 frames/second. The test video is displayed and the DVS captures that. The analysis is performed at the instant the circle approaches the center of the display. As we have extracted the transformation matrix from real coordinates to camera coordinates, we know the exact position of the circle center and we can calculate events distance from the circle center. Figure 14 shows the circle before and after applying the extended event-based Hough transform algorithm. The plotted events' distance distribution from the circle center presented in Figure 14, shows the distribution is narrower around 5*cm* (circle radius) after cleaning the video. The circle boundary is recognized as a series of small lines. The lines distance from the circle center is 5.10±0.16*cm*. Moreover, the error of these lines orientation with respect to the ground truth is 3^*o*^. In future, we use extracted line elements to detect any arbitrary shapes and their parameters e.g., curvature.

## 8. Conclusion

In this paper we systematically discussed efficient implementation of Hough Transform (HT) for event-based systems, the concepts that should be considered for each application, and the challenges. We discussed DVS as the hardware basis of our system, and how HT has interesting intrinsic properties suitable for image processing on such event-based systems. As the Standard HT (SHT) cannot be readily applied to event-based systems, as it is structured for frame-based input streams (i.e. conventional cameras). We firstly discussed an extended HT, adopting of a specific third dimension for the parameter space, can address multiple shortcomings of SHT, and make it suitable to be implemented for event-based systems. We also discussed an intrinsic problem of SHT, the non-uniformity of projection between the Cartesian space and the parameter space, that leads to several further challenges in implementation and usage of HT, and how this intrinsic problem can be solved using this third dimension. The described extended 3D HT significantly improve application of HT by allowing detection of line segments with specific length and location, detecting end points of each segment, differentiating noise and real line segments, and extracting important shape information such as locally linear components and curvature. We then moved to describe how Spiking Neural Networks (SNNs) can implement the parameter space of 2D and 3D HT. SNNs can be easily implemented in hardware via FPGA and therefore the entire 3D HT can be performed on the output of a DVS camera, with no requirement for CPU or additional memory. Furthermore, the increase in complexity of the shape, or accuracy over a specific parameter does not increase the computational complexity and the entire transformation and detection can be still implemented in FPGA. With the extended 3D HT in picture, we then discussed the main aspects of SNNs for different applications including thresholding, decay rate, network inhibitory connections in space and time, and parameter space quantization. While we discussed the most important aspects and pitfalls for each of these concepts, each one of the parameters can be investigated in significantly more details for a specific set of applications.

The proposed method without any further post-processing is useful to extract the framework of objects in event-based videos and remove those events caused by either noise or the complex texture of the object. The advantage of this algorithm compared to other similar algorithms for cleaning noise is that the amount of details which is removed is completely controllable. The algorithm keeps only those events that can potentially create a small line segment and omits all other events. As a result, the stream retains only those events which most likely come from a real edge of an object, not the texture details of that.

If we aim to detect a shape, the proposed method will need a post-processing layer as well. In applications like shape encoding, this layer should perform an exhaustive search in the 3D parameter space to find any arbitrary shapes. Although to the best of our knowledge, in most applications e.g., shape detection, a presumption on the desired shape is usually needed. If the algorithm is meant to find a particular shape in the stream, the search area in parameter space will be highly limited. For example, the search area for detecting all line segments in a frame, is vertical lines in parameter space which have a fixed *r* and *θ*. In other words, each line segment in Cartesian space generates some spikes from successive spiking neurons which are in red color in Figure 5. It is noted that the post-processing for detecting shapes as a part of our future work is out of this manuscript scope.

We hope that this comprehensive discussion can help researchers in understanding the underlying factors, avoid pitfalls, and adapting suitable parameters to meet the need of their applications. We also hope that through extended use of Hough Transform, computer vision approaches applicable to event-based systems can be elevated to a higher level of complexity, closing the gap between these systems and conventional frame-based cameras.

Some possible future works include shape encoding through projection of the template to the 3D HT, invariant template matching given the encoded shape, and detection and tracking of object in multi-views.

## Author Contributions

W-YY initiated and supervised the research. SS designed and implemented the algorithms. HN and SS wrote the manuscript. Both W-YY and KM reviewed and revised the draft. HN improved some parts.

### Conflict of Interest Statement

The authors declare that the research was conducted in the absence of any commercial or financial relationships that could be construed as a potential conflict of interest.

## References

[B1] Achalakul T. Madarasmi S. (2002). A concurrent modified algorithm for Generalized Hough Transform in IEEE International Conference on Industrial Technology, Vol. 2, (Bankok), 965–969. 10.1109/ICIT.2002.1189300

[B2] Arnold R. Miklós P. (2010). Character recognition using neural networks in 11th IEEE International Symposium on Computational Intelligence and Informatics, CINTI 2010 - Proceedings (Budapest), 311–314.

[B3] Bachiller-Burgos P. Manso L. J. Bustos P. (2018). A spiking neural model of HT3D for corner detection. Front. Comput. Neurosci. 12:37. 10.3389/fncom.2018.00037 29910720 PMC5992504

[B4] Ballard D. H. (1981). Generalizing the Hough transform to detect arbitrary shapes. Patt. Recogn. 13, 111–122. 10.1016/0031-3203(81)90009-1

[B5] Bauer D. Belbachir A. N. Donath N. Gritsch G. Kohn B. Litzenberger M. (2007). Embedded vehicle speed estimation system using an asynchronous temporal contrast vision sensor. EURASIP J. Embed. Syst. 2007, 1–12. 10.1155/2007/82174

[B6] Beinglass A. Wolfson H. (1991). Articulated object recognition, or: how to generalize the generalized Hough transform in IEEE Computer Society Conference on Computer Vision and Pattern Recognition (Maui, HI), 461–466.

[B7] Benosman R. Clercq C. Lagorce X. Ieng S. H. Bartolozzi C. (2014). Event-based visual flow. IEEE Trans. Neural Netw. Learn. Syst. 25, 407–417. 10.1109/TNNLS.2013.2273537 24807038

[B8] Benosman R. Ieng S. H. Clercq C. Bartolozzi C. Srinivasan M. (2012). Asynchronous frameless event-based optical flow. Neural Netw. 27, 32–37. 10.1016/j.neunet.2011.11.001 22154354

[B9] Bergen J. R. Shvaytser (Schweitzer) H. (1991). A probabilistic algorithm for computing Hough transforms. J. Algorithms 12, 639–656. 10.1016/0196-6774(91)90037-Y

[B10] Berner R. Brandli C. Yang M. Liu S. C. (2013). A 240-by-180 10mW 12us latency sparse-output vision sensor for mobile applications 2013 Symposium on VLSI Circuits (VLSIC) (Kyoto), 186–187. 10.23919/MEASUREMENT.2017.7983527

[B11] Bonnet N. (2002). An unsupervised generalized Hough transform for natural shapes. Patt. Recogn. 35, 1193–1196. 10.1016/S0031-3203(01)00219-9

[B12] Brandli C. Berner R. Yang M. Liu S. C. Delbruck T. (2014). A 240 X 180 130 dB 3 μs latency global shutter spatiotemporal vision sensor. IEEE J. Solid State Circ. 49, 2333–2341. 10.1109/JSSC.2014.2342715

[B13] Brandli C. Mantel T. A. Hutter M. Höpflinger M. A. Berner R. Siegwart R. Delbruck T. (2013). Adaptive pulsed laser line extraction for terrain reconstruction using a dynamic vision sensor. Front. Neurosci. 7:275. 10.3389/fnins.2013.00275 24478619 PMC3894568

[B14] Bruckmann A. Klefenz F. Wunsche A. (2014). A neural net for 2D-slope and sinusoidal shape detection. Int. J. Comput. 3, 21–26. Available online at: http://computingonline.net/computing/article/view/248

[B15] Burkitt A. N. (2006a). A review of the integrate-and-fire neuron model: I. Homogeneous synaptic input. Biol. Cybernet. 95, 1–19. 10.1007/s00422-006-0068-6 16622699

[B16] Burkitt A. N. (2006b). A review of the integrate-and-fire neuron model: II. Inhomogeneous synaptic input and network properties. Biol. Cybernet. 95, 97–112. 10.1007/s00422-006-0082-8 16821035

[B17] Chan R. (1991). New parallel Hough transform for circles. IEE Proc. Comput. Digital Techn. 138, 335–344. 10.1049/ip-e.1991.0046

[B18] Chatzis V. Pitas I. (1996). Select and split fuzzy cell Hough transform-a fast and efficient method to detect contours in images in IEEE 5th International Fuzzy Systems (New Orleans, LA), 1892–1898. 10.1109/FUZZY.1996.552687

[B19] Chatzis V. Pitas I. (1997). Randomized fuzzy cell Hough transform in Proceedings of 6th International Fuzzy Systems Conference (Barcelona: IEEE>), 1185–1190. 10.1109/FUZZY.1997.622876

[B20] Chau C. P. Siu W. C. (2004). Adaptive dual-point Hough transform for object recognition. Comput. Vis. Image Understand. 96, 1–16. 10.1016/j.cviu.2004.04.005

[B21] Chen S. Akselrod P. Zhao B. Carrasco J. A. P. Linares-Barranco B. Culurciello E. (2012). Efficient feedforward categorization of objects and human postures with address-event image sensors. IEEE Trans. Patt. Anal. Mach. Intell. 34, 302–314. 10.1109/TPAMI.2011.120 21670481

[B22] Clady X. Clercq C. Ieng S. H. Houseini F. Randazzo M. Natale L. Bartolozzi C. Benosman R. (2014). Asynchronous visual event-based time-to-contact. Front. Neurosci. 8:9. 10.3389/fnins.2014.00009 24570652 PMC3916774

[B23] Clady X. Ieng S. H. Benosman R. (2015). Asynchronous event-based corner detection and matching. Neural Netw. 66, 91–106. 10.1016/j.neunet.2015.02.013 25828960

[B24] Davies E. R. (1986). Image space transforms for detecting straight edges in industrial images. Patt. Recogn. Lett. 4, 185–192. 10.1016/0167-8655(86)90018-8

[B25] Delbruck T. Lang M. (2013). Robotic goalie with 3 ms reaction time at 4% CPU load using event-based dynamic vision sensor. Front. Neurosci. 7:223. 10.3389/fnins.2013.00223 24311999 PMC3836084

[B26] Delbruck T. Lichtsteiner P. (2007). Fast sensory motor control based on event-based hybrid neuromorphic-procedural system. IEEE International Symposium on Circuits and Systems (New Orleans, LA), 845–848. 10.1109/ISCAS.2007.378038

[B27] Duda R. O. and Hart, P. E. (1972). Use of the Hough transformation to detect lines and curves in pictures. Commun. ACM 15, 11–15. 10.1145/361237.361242

[B28] Dyer C. R. (1983). Gauge inspection using Hough transforms in IEEE Transactions on Pattern Analysis and Machine Intelligence, PAMI-5(6), 621–623. 10.1109/TPAMI.1983.4767452 21869149

[B29] Epstein A. Boulin C. Paul G. U. Vettermann B. Klefenz F. (2002). A parallel systolic array ASIC for real-time execution of the Hough transform. IEEE Trans. Nuclear Sci. 49, 339–346. 10.1109/TNS.2002.1003733

[B30] Fei H. Yanling G. Lili W. (2009). A new ellipse detector based on Hough transform in 2nd International Conference on Information and Computing Science, ICIC 2009, ICIC '09 (Washington, DC: IEEE Computer Society), 301–305.

[B31] Galamhos C. Matas J. Kittler J. (1999). Progressive probabilistic Hough transform for line detection in IEEE Computer Society Conference on Computer Vision and Pattern Recognition (Cat. No PR00149) (Fort Collins, CO), 554–560. 10.1109/CVPR.1999.786993

[B32] Gatos B. Perantonis S. J. Papamarkos N. (1996). Accelerated Hough transform using rectangular image decomposition. Electron. Lett. 32, 730–732. 10.1049/el:19960510

[B33] Gerstner W. Kistler W. M. Naud R. Paninsk L. (2014). From Single Neurons to Networks and Models of Cognition. Cambridge University Press Available online at: http://neuronaldynamics.epfl.ch/online/Ch1.S3.html

[B34] Ghosh-dastidar S. Lichtenstein A. G. (2009). Spiking neural networks. Int. J. Neural Syst. 19, 295–308. 10.1142/S0129065709002002 19731402

[B35] Gollisch T. Meister M. (2008). Rapid neural coding in the retina with relative spike latencies. Science 319, 1108–1111. 10.1126/science.1149639 18292344

[B36] Gütig R. Sompolinsky H. (2006). The tempotron: a neuron that learns spike timing-based decisions. Nat. Neurosci. 9, 420–428. 10.1038/nn1643 16474393

[B37] Hakalahti H. Harwood D. Davis L. S. (1984). Two-dimensional object recognition by matching local properties of contour points. Patt. Recogn. Lett. 2, 227–234. 10.1016/0167-8655(84)90029-1

[B38] Hodgkin A. L. Huxley A. F. (1990). A quantitative description of membrane current and its application to conduction and excitation in nerve. Bull. Math. Biol. 52, 25–71. 10.1007/BF02459568 2185861

[B39] Hough P. V. C. (1962). Method and Means for Recognizing Complex Patterns. US patent 3069654. United States Patent Office. Available online at: http://www.freepatentsonline.com/3069654.html

[B40] Hu J. Tang H. Tan K. C. Li H. Shi L. (2013). A spike-timing-based integratedmodel for pattern recognition. Neural Comput. 25, 450–472. 10.1162/NECO_a_00395 23148414

[B41] Illingworth J. Kittler J. (1987). The adaptive Hough transform in IEEE Transactions on Pattern Analysis and Machine Intelligence, PAMI-9(5) (IEEE Computer Society), 690–698. 10.1109/TPAMI.1987.4767964 21869428

[B42] Ioannou D. Huda W. Laine A. F. (1999). Circle recognition through a 2D Hough Transform and radius histogramming. Image Vis. Comput. 17, 15–26. 10.1016/S0262-8856(98)00090-0

[B43] Izhikevich E. M. (2003). Simple model of spiking neurons. IEEE Trans. Neural Netw. 14, 1569–1572. 10.1109/TNN.2003.820440 18244602

[B44] Izhikevich E. M. (2004). Which model to use for cortical spiking neurons? IEEE Trans. Neural Netw. 15, 1063–1070. 10.1109/TNN.2004.832719 15484883

[B45] Jeng S. C. Tsai W. H. (1991). Scale- and orientation-invariant generalized hough transform3-a new approach. Patt. Recogn. 24, 1037–1051. 10.1016/0031-3203(91)90120-T

[B46] Jonas P. Buzsaki G. (2007). Neural inhibition. Scholarpedia 2:3286 10.4249/scholarpedia.3286

[B47] Jung C. R. Schramm R. (2004). Rectangle detection based on a windowed hough transform in Brazilian Symposium of Computer Graphic and Image Processing (IEEE Computer Society), 113–120. 10.1109/SIBGRA.2004.1352951

[B48] Kang S. K. Choung Y. C. Park J. A. (2005). Image corner detection using Hough transform in Pattern Recognition and Image Analysis, eds Marques J. S. Prez de la Blanca N. Pina P. (Berlin; Heidelberg: Springer), 279–286.

[B49] Kasabov N. Feigin V. Hou Z. G. Chen Y. Liang L. Krishnamurthi R. (2014). Evolving spiking neural networks for personalised modelling, classification and prediction of spatio-temporal patterns with a case study on stroke. Neurocomputing 134, 269–279. 10.1016/j.neucom.2013.09.049

[B50] Kerbyson D. (1995). Circle detection using Hough transform filters in Fifth International Conference on Image Processing and Its Applications (Edinburgh), 370–374. 10.1049/cp:19950683

[B51] Kimura A. Watanabe T. (2002). An extension of the generalized Hough transform to realize affine-invariant two-dimensional (2D) shape detection in 16th International Conference on Pattern Recognition (ICPR'02) (Washington, DC: IEEE Computer Society), 1–4.

[B52] Klefenz F. Noffz K.-H. Conen W. Zoz R. Kugel A. Manner R. (1993). Track recognition in 4 ms by a systolic trigger processor using a parallel Hough transform IEEE Conference on Nuclear Science Symposium and Medical Imaging (Orlando, FL), 302–304. 10.1109/NSSMIC.1992.301235

[B53] Kohn B. Belbachir A. N. Hahn T. Kaufmann H. (2012). Event-driven body motion analysis for real-time gesture recognition in IEEE International Symposium on Circuits and Systems (Seoul: IEEE), 703–706. 10.1109/ISCAS.2012.6272132

[B54] Koshimizu H. Numada M. (1990). On a fast Hough transform method PLHT based on piecewise-linear Hough function. Syst. Comput. Japan 21, 62–73. 10.1002/scj.4690210506

[B55] Kröger B. J. Kannampuzha J. Lowit A. Neuschaefer-Rube C. (2009). Phonetotopy within a neurocomputational model of speech production and speech acquisition. Some Aspect Speech Brain 51, 59–90. 10.1016/j.specom.2008.08.002

[B56] Lagorce X. Meyer C. Ieng S. H. Filliat D. Benosman R. (2015). Asynchronous event-based multikernel algorithm for high-speed visual features tracking. IEEE Trans. Neural Netw. Learn. Syst. 26, 1710–1720. 10.1109/TNNLS.2014.2352401 25248193

[B57] Laing C. R. Longtin A. (2003). Dynamics of deterministic and stochastic paired excitatory-inhibitory delayed feedback. Neural Comput. 15, 2779–2822. 10.1162/089976603322518740 14629868

[B58] Leavers V. F. (1990). Active intelligent vision using the dynamic generalized Hough transform in British Machine Vision Conference 1990 (Oxford, UK), 11.1–11.6. 10.5244/C.4.11

[B59] Leñero-Bardallo J. A. Serrano-Gotarredona T. Linares-Barranco B. (2011). A 3.6 μ s latency asynchronous frame-free event-driven dynamic-vision-sensor. IEEE J. Solid State Circ. 46, 1443–1455. 10.1109/JSSC.2011.2118490

[B60] Li H. Lavin M. A. Le Master R. J. (1986). Fast Hough transform: a hierarchical approach. Comput. Vis. Graph. Image Process. 36, 139–161. 10.1016/0734-189X(86)90073-3

[B61] Lichtsteiner P. Posch C. Delbruck T. (2006). A 128 X 128 120db 30mw asynchronous vision sensor that responds to relative intensity change in IEEE International Solid State Circuits Conference - Digest of Technical Papers (San Francisco, CA: IEEE), 2060–2069. 10.1109/ISSCC.2006.1696265

[B62] Lichtsteiner P. Posch C. Delbruck T. (2008). A 128 X 128 120 dB 15 us latency asynchronous temporal contrast vision sensor. IEEE J. Solid State Circ. 43, 566–576. 10.1109/JSSC.2007.914337

[B63] Lo R. C. Tsai W. H. (1997). Perspective-transformation-invariant generalized hough transform for perspective planar shape detection and matching. Patt. Recogn. 30, 383–396.

[B64] López-González G. Altamirano-Gómez G. Bayro-Corrochano E. (2016). Geometric entities voting schemes in the conformal geometric algebra framework. Adv. Appl. Clifford Algebras 26, 1045–1059. 10.1007/s00006-015-0589-y

[B65] Martin K. A. (2006). Encyclopedia of Cognitive Science. American Cancer Society.

[B66] Meftah B. Lezoray O. Benyettou A. (2010). Segmentation and edge detection based on spiking neural network model. Neural Process. Lett. 32, 131–146. 10.1007/s11063-010-9149-6

[B67] Merlin P. M. Farber D. J. (1975). A parallel mechanism for detecting curves in pictures. IEEE Trans. Comput. C-24, 96–98. 10.1109/T-C.1975.224087

[B68] Merolla P. A. Arthur J. V. Alvarez-Icaza R. Cassidy A. S. Sawada J. Akopyan F.. (2014). A million spiking-neuron integrated circuit with a scalable communication network and interface. Science 345, 668–673. 10.1126/science.1254642 25104385

[B69] Muammar H. Nixon M. (1989). Approaches to extending the Hough transform in International Conference on Acoustics, Speech, and Signal Processing (Glasgow), 1556–1559. 10.1109/ICASSP.1989.266739

[B70] Neftci E. Das S. Pedroni B. Kreutz-Delgado K. Cauwenberghs G. (2013). Event-driven contrastive divergence for spiking neuromorphic systems. Front. Neurosci. 7:272. 10.3389/fnins.2013.00272 24574952 PMC3922083

[B71] Ni Z. Pacoret C. Benosman R. Ieng S. Régnier S. (2012). Asynchronous event-based high speed vision for microparticle tracking. J. Microsc. 245, 236–244. 10.1111/j.1365-2818.2011.03565.x

[B72] Olson C. F. (1999). Constrained Hough transforms for curve detection. Computer .Vis. Image Understand. 73, 329–345.

[B73] Painkras E. Plana L. A. Garside J. Temple S. Galluppi F. Patterson C. (2013). SpiNNaker: a 1-W 18-core system-on-chip for massively-parallel neural network simulation. IEEE J. Solid State Circ. 48, 1943–1953. 10.1109/JSSC.2013.2259038

[B74] Pao D. C. Li H. F. Jayakumar R. (1992). Shapes recognition using the straight line Hough transform: theory and generalization. IEEE Trans. Patt. Anal. Mach. Intell. 14, 1076–1089. 10.1109/34.166622

[B75] Pérez-Carrasco J. A. Zhao B. Serrano C. Acha B. Serrano-Gotarredona T. Chen S.. (2013). Mapping from frame-driven to frame-free event-driven vision systems by low-rate rate coding and coincidence processing–application to feedforward ConvNets. IEEE Trans. Patt. Anal. Mach. Intell. 35, 2706–2719. 10.1109/TPAMI.2013.71 24051730

[B76] Ponulak F. Kasiński A. (2010). Supervised learning in spiking neural networks with ReSuMe: Sequence learning, classification, and spike shifting. Neural Comput. 22, 467–510. 10.1162/neco.2009.11-08-901 19842989

[B77] Posch C. Serrano-Gotarredona T. Linares-Barranco B. Delbruck T. (2014). Retinomorphic event-based vision sensors: bioinspired cameras with spiking output. Proc. IEEE 102, 1470–1484. 10.1109/JPROC.2014.2346153

[B78] Rad A. A. Faez K. Qaragozlou N. (2003). Fast circle detection using gradient pair vectors in 7th Digital Image Computing: Techniques and Applications (Sydney, NSW), 879–887.

[B79] Samal A. Edwards J. (1997). Generalized Hough transform for natural shapes. Patt. Recogn. Lett. 18, 473–480. 10.1016/S0167-8655(97)00023-8

[B80] Sanz J. L. C. Dinstein I. Petkovic D. (1987). Computing multi-colored polygonal masks in pipeline architecture and its application to automated visual inspection. Commun. ACM 30, 318–329. 10.1145/32232.32235

[B81] Scaramuzza R. D. Floreano U. (2014). High-Speed Pose Estimation Using a Dynamic Vision Sensor. Master thesis, University of Zurich.

[B82] Seifozzakerini S. Yau W.-Y. Mao K. (2017). Effect of inhibitory window on event-based Hough transform for multiple lines detection in International Conference on Advances in Image Processing-ICAIP 2017 (New York, NY: ACM Press), 39–44.

[B83] Seifozzakerini S. Yau W.-Y. Zhao B. Mao K. (2016). Event-based Hough transform in a spiking neural network for multiple line detection and tracking using a dynamic vision sensor in British Machine Vision Conference 2016 (York), 94.1–94.12. 10.1016/S0140-6736(86)92160-4

[B84] Ser P.-K. Siu W.-C. (1992). Sampling Hough algorithm for the detection of lines and curves in IEEE International Symposium on Circuits and Systems (San Diego, CA), 2497–2500. 10.1109/ISCAS.1992.230479

[B85] Ser P.-K. Siu W.-C. (1995). A new generalized Hough transform for the detection of irregular objects. J. Vis. Commun. Image Represent. 6, 256–264. 10.1006/jvci.1995.1022

[B86] Serrano-Gotarredona T. Linares-Barranco B. (2013). A 128 X 128 1.5% contrast sensitivity 0.9% FPN 3 us latency 4 mW asynchronous frame-free dynamic vision sensor using transimpedance preamplifiers. IEEE J. Solid State Circ. 48, 827–838. 10.1109/JSSC.2012.2230553

[B87] Shen F. Wang H. (2002). Corner detection based on modified Hough transform. Patt. Recogn. Lett. 23, 1039–1049. 10.1016/S0167-8655(02)00035-1

[B88] Smith L. (2010). Neuromorphic systems: past, present and future. Brain Inspired Cogn. Syst. 657, 167–182. 10.1007/978-0-387-79100-5_9 20020347

[B89] Soria-García G. Altamirano-Gómez G. Ortega-Cisneros S. Bayro-Corrochano E. (2017). FPGA implementation of a geometric voting scheme for the extraction of geometric entities from images. Adv. Appl. Clifford Algebras 27, 685–705. 10.1007/s00006-016-0708-4

[B90] Tavanaei A. Maida A. (2017). Bio-inspired multi-layer spiking neural network extracts discriminative features from speech signals in Neural Information Processing, eds Liu D. Xie S. Li Y. Zhao D. El-Alfy E. S. M. (Cham: Springer International Publishing), 899–908.

[B91] Tsai D.-M. (1997). An improved generalized Hough transform for the recognition of overlapping objects. Image Vis. Comput. 15, 877–888. 10.1016/S0262-8856(97)00033-4

[B92] Tsuji S. Matsumoto F. (1978). Detection of ellipses by a modified Hough transformation. IEEE Trans. Comput. C-27, 777–781. 10.1109/TC.1978.1675191

[B93] Valeiras D. R. Lagorce X. Clady X. Bartolozzi C. Ieng S.-H. Benosman R. (2015). An asynchronous neuromorphic event-driven visual part-based shape tracking. IEEE Trans. Neural Netw. Learn. Syst. 26, 3045–3059. 10.1109/TNNLS.2015.2401834 25794399

[B94] Wade J. J. McDaid L. J. Santos J. A. Sayers H. M. (2010). SWAT: a spiking neural network training algorithm for classification problems. IEEE Trans. Neural Netw. 21, 1817–1830. 10.1109/TNN.2010.2074212 20876015

[B95] Wallace A. M. (1983). Greyscale image processing for industrial applications. Image Vis. Comput. 1, 178–188.

[B96] Weikersdorfer D. Conradt J. (2012). Event-based particle filtering for robot self-localization in IEEE International Conference on Robotics and Biomimetics, ROBIO 2012 - Conference Digest (Guangzhou: IEEE), 866–870. 10.1109/ROBIO.2012.6491077

[B97] Weikersdorfer D. Hoffmann R. Conradt J. (2013). Simultaneous localization and mapping for event-based vision systems Computer Vision Systems, eds Chen M. Leibe B. Neumann B. (Berlin; Heidelberg: Springer), 133–142. 10.1007/978-3-642-39402-7

[B98] Wysoski S. G. Benuskova L. Kasabov N. (2010). Evolving spiking neural networks for audiovisual information processing. Neural Netw. 23, 819–835. 10.1016/j.neunet.2010.04.009 20510579

[B99] Xu L. Oja E. Kultanen P. (1990). A new curve detection method: randomized Hough transform (RHT). Patt. Recogn. Lett. 11, 331–338. 10.1016/0167-8655(90)90042-Z

[B100] Yu Q. Tang H. Tan K. C. Li H. (2013a). Precise-spike-driven synaptic plasticity: learning hetero-association of spatiotemporal spike patterns. PLoS ONE 8:e78318. 10.1371/journal.pone.0078318 24223789 PMC3818323

[B101] Yu Q. Tang H. Tan K. C. Li H. (2013b). Rapid feedforward computation by temporal encoding and learning with spiking neurons. IEEE Trans. Neural Netw. Learn. Systems 24, 1539–1552. 10.1109/TNNLS.2013.2245677 24808592

[B102] Yuen H. K. Illingworth J. Kittler J. (1989). Detecting partially occluded ellipses using the Hough transform. Image Vis. Comput. 7, 31–37. 10.1016/0262-8856(89)90017-6

[B103] Zhao B. Ding R. Chen S. Linares-Barranco B. Tang H. (2015). Feedforward categorization on AER motion events using cortex-like features in a spiking neural network. IEEE Trans. Neural Netw. Learn. Syst. 26, 1963–1978. 10.1109/TNNLS.2014.2362542 25347889

[B104] Zhao B. Zhang X. Chen S. Low K.-S. Zhuang H. (2012). A 64 X 64 CMOS image sensor with on-chip moving object detection and localization. IEEE Trans. Circ. Syst. Video Technol. 22, 581–588. 10.1109/TCSVT.2011.2170119

